# Primary and metastatic tumors exhibit systems-level differences in dependence on mitochondrial respiratory function

**DOI:** 10.1371/journal.pbio.3001753

**Published:** 2022-09-22

**Authors:** Neal K. Bennett, Hiroki J. Nakaoka, Danny Laurent, Ross A. Okimoto, Yoshitaka Sei, Andrew E. Horvai, Trever G. Bivona, Johanna ten Hoeve, Thomas G. Graeber, Ken Nakamura, Jean L. Nakamura

**Affiliations:** 1 Gladstone Institute of Neurological Disease, Gladstone Institutes, San Francisco, California, United States of America; 2 Department of Radiation Oncology, University of California, San Francisco, San Francisco, California, United States of America; 3 Department of Medicine, University of California, San Francisco, San Francisco, California, United States of America; 4 Department of Pathology, University of California, San Francisco, San Francisco, California, United States of America; 5 Department of Molecular and Medical Pharmacology, Crump Institute for Molecular Imaging, UCLA Metabolomics Center, University of California, Los Angeles, Los Angeles, California, United States of America; 6 Graduate Programs in Neuroscience and Biomedical Sciences, University of California, San Francisco, San Francisco, California, United States of America; 7 Department of Neurology, University of California, San Francisco, San Francisco, California, United States of America; University of California Los Angeles, UNITED STATES

## Abstract

The Warburg effect, aerobic glycolysis, is a hallmark feature of cancer cells grown in culture. However, the relative roles of glycolysis and respiratory metabolism in supporting in vivo tumor growth and processes such as tumor dissemination and metastatic growth remain poorly understood, particularly on a systems level. Using a CRISPRi mini-library enriched for mitochondrial ribosomal protein and respiratory chain genes in multiple human lung cancer cell lines, we analyzed in vivo metabolic requirements in xenograft tumors grown in distinct anatomic contexts. While knockdown of mitochondrial ribosomal protein and respiratory chain genes (mito-respiratory genes) has little impact on growth in vitro, tumor cells depend heavily on these genes when grown in vivo as either flank or primary orthotopic lung tumor xenografts. In contrast, respiratory function is comparatively dispensable for metastatic tumor growth. RNA-Seq and metabolomics analysis of tumor cells expressing individual sgRNAs against mito-respiratory genes indicate overexpression of glycolytic genes and increased sensitivity of glycolytic inhibition compared to control when grown in vitro, but when grown in vivo as primary tumors these cells down-regulate glycolytic mechanisms. These studies demonstrate that discrete perturbations of mitochondrial respiratory chain function impact in vivo tumor growth in a context-specific manner with differential impacts on primary and metastatic tumors.

## Abstract 2

This study shows that primary and metastatic tumors have discrete requirements for glycolysis- or respiration-derived ATP, suggesting that tumors growing in different anatomic sites develop metabolic heterogeneity, asymmetrically depending upon glycolysis or respiratory metabolism.

## Introduction

The dysregulation of cellular energy metabolism is an early fundamental event in tumorigenesis and a hallmark of cancer. Cancer cells modulate their metabolism as they proliferate, outpace normal cells in growth, and establish disease in diverse and often nutrient-restricted environments. The Warburg effect observed in cancer cells refers to the preferential use of aerobic glycolysis, which produces less ATP than aerobic respiration while favoring biosynthetic functions necessary for tumor growth [1]. The specific roles of ATP-modulating mechanisms in supporting tumor growth are poorly understood, particularly in vivo. Most studies investigating respiration and the Warburg effect are performed in cultured cells. While these have established that mitochondria are necessary for tumorigenesis [2] and have led to cancer therapies that target oxidative phosphorylation [3], precisely how mitochondria participate in cancer metabolic programs is still poorly understood, especially as it pertains to in vivo tumor growth in different anatomic and microenvironmental contexts. In contrast to in vitro tumor modeling, tumor growth in vivo tests specific physiologic contexts in which cancer cells must metabolically adapt to thrive. Metabolomics analyses of lung cancers growing in vivo indicate increased glucose-derived carbon-labeling of TCA intermediates compared to normal lung [4], supporting the hypothesis that tumor metabolism and fuel utilization are distinct from those of normal tissues. However, how tumors and metastases grow in diverse anatomic locations, and the identity of the metabolic programs underpinning this capacity, are poorly understood.

In prior work, we developed a high-throughput screening paradigm to identify genetic regulators of ATP, combining FACS and CRISPR with an ATP-FRET sensor capable of monitoring real-time changes in ATP concentrations within individual living cells [5]. Using this sensor, we performed genome-wide CRISPRi and CRISPRa screens to define an “ATPome” of genes and pathways that regulate ATP levels through energy substrate-specific pathways (respiration or glycolysis) [6]. A key finding from this work is that many genes and pathways that preserve or reduce ATP exert these effects only *under specific metabolic conditions defined by substrate availability*. Glycolysis and respiration demonstrate cross-optimization on a systems level, that is, suppression of 1 metabolic mechanism (via members of specific gene classes) optimizes the alternative mechanism. Silencing genes required for respiratory-derived ATP modestly suppressed in vitro growth under respiratory conditions but conversely increased in vitro tumor cell growth under glycolytic conditions. This evidence of cross-optimization points adaptive metabolism that is measurable in cellular ATP; it also indicates a broader repertoire of mechanisms available to cells for optimizing their metabolic function.

Whether and how respiration and glycolysis-derived ATP production differentially impact in vivo growth, especially when multiple anatomically separate tumor sites have developed, is not known on a systems level. To evaluate this, we developed a custom CRISPRi mini-library composed of sgRNAs against genes modulating cellular ATP levels (identified from the ATPome [6]), to test whether ATP levels correlate with in vivo growth of tumors in multiple preclinical mouse models. In vivo xenograft models revealed differential growth effects of ATP-modulating genes within discrete anatomic sites, where suppression of genes involved in mitochondrial-derived ATP was associated with reduced growth of primary tumors but not their associated metastases. To evaluate the molecular basis for this reduced growth, we performed RNA Seq and metabolomics analysis in isogenic lung cancer cells engineered with discrete silencing of mito-respiratory genes; these studies indicated profound adaptive metabolism, particularly involving the glycolytic metabolic profile, which was highly dependent on the tumor growth context. Targeting genes that modulate ATP level in parallel models identified discrete systems-level requirements for mitochondrial and respiratory chain function in primary versus metastatic tumors, illustrating asymmetric dependence on glycolysis or respiratory metabolism, indicative of metabolic heterogeneity.

To our knowledge, a systems-level analysis of genetic modulators of ATP combined with in vivo functional readouts has not been performed in human cancers. By evaluating tumor growth in multiple in vivo contexts, our findings illustrate critical differences in the mitochondrial respiratory chain requirements between primary and metastatic tumor growth, pointing to a functional role for respiratory chain function that is specific to regional growth and distinguishes primary tumors from distant metastases.

## Results

### Primary in vivo tumor growth requires mitochondrial-derived ATP

Lung cancer is the most common cause of death due to cancer in the United States and is characterized by primary solid tumors that can metastasize widely to diverse organs. HCC827 and H1975 human lung cancer cells are model cell lines for epidermal growth factor receptor (EGFR)-mutant nonsmall cell lung cancer [7,8]. To determine whether ATP-modulating genes impact the in vivo tumor growth of lung cancers, we transduced HCC827 cells expressing dCas9-KRAB with a custom CRISPRi mini-library enriched with respiratory and glycolytic hits that most significantly influenced ATP levels (high or low, depending on the substrate conditions) in a previous screen in K562 and HCC827 cells [6]. This mini-library contains over 400 sgRNAs (1/5 of which are nontargeting sgRNAs included as negative controls), with multiple sgRNAs against each gene target (typically 2 to 4 unique sgRNAs/gene) [6].

**Fig 1:**
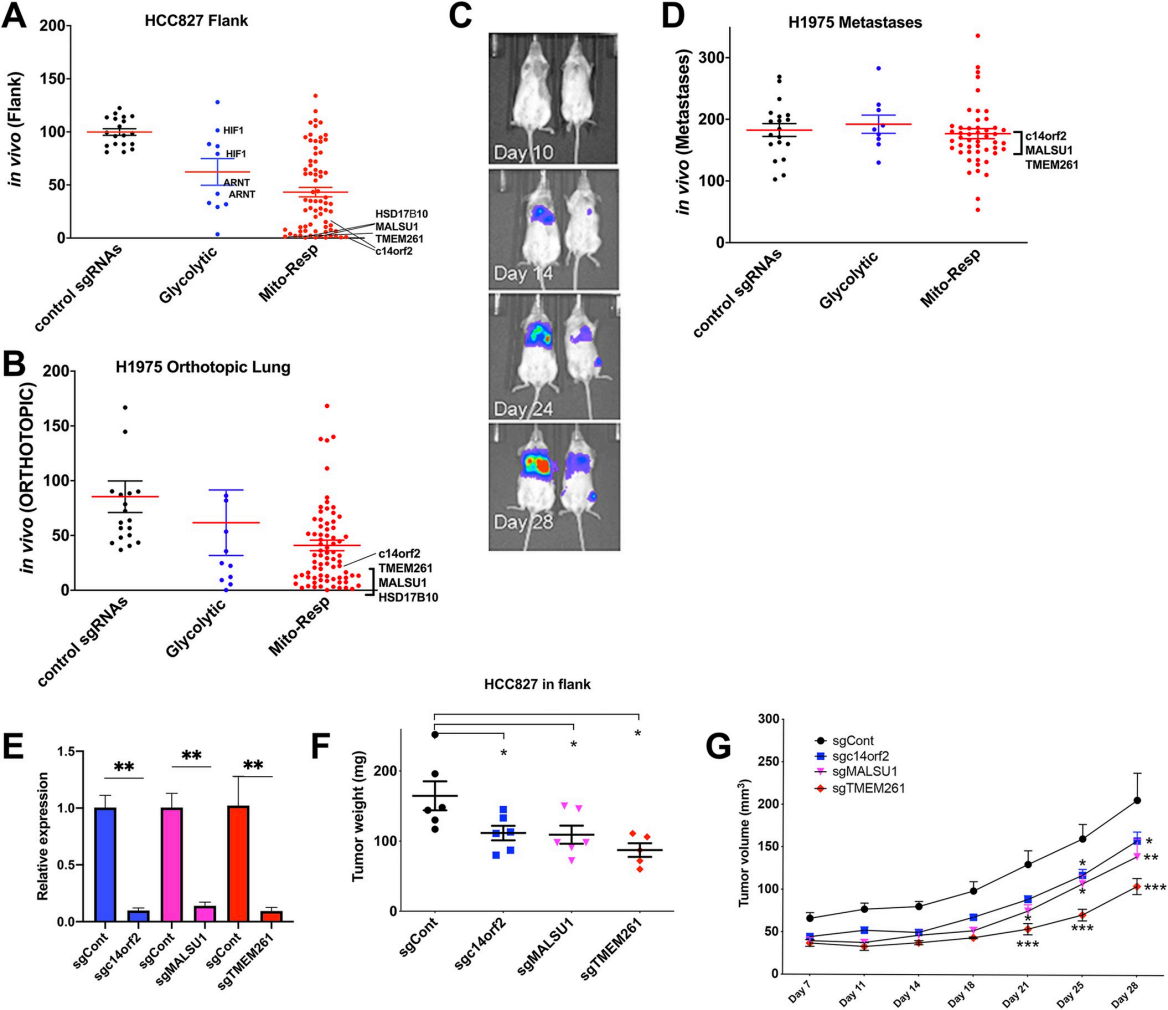
Human lung cancer cells demonstrate differential requirements for mitochondrial and respiratory genes when grown in vivo in flank or orthotopic lung models. HCC827 and H1975 human *EGFR*-mutant lung cancer cells were transduced with the mini-CRISPRi library and then injected into either the subcutaneous space in flanks (HCC827) or the left lower lung lobes (H1975) of nude mice and grown for 28 days. DNA from each tumor was sequenced and read counts for each sgRNA quantified. The read count for each sgRNA was normalized to the sum of reads for negative control sgRNAs and the ratio of each sgRNA’s frequency in the tumor model relative to its frequency in vitro (immediately preinjection). (**A)** Read count frequency in the HCC827 cell model. Two independent replicate experiments were performed, with *n* = 6 for each experiment (for *n* = 12 total). Each dot represents a single sgRNA and indicates its average normalized representation (computed from 2 independent replicate experiments), organized and displayed as control sgRNAs and sgRNAs targeting ATP-modulating genes classified as glycolytic or mito-respiratory (termed Mito-Resp). Mito-Resp sgRNAs were among the most severely depleted in vivo (*HSD17β10*, *TMEM261*, *MALSU1*, *c14orf2*) (mean and SEM shown) (mean % representation of nontargeting, glycolytic and mito-respiratory sgRNAs as groups are 99.9%, 62.6%, and 42.2%, respectively. One-way ANOVA of all 3 groups of sgRNAs demonstrate *p*-value < 0.0001, with Tukey’s multiple comparisons test between glycolytic and control *p* < 0.02, mito-resp and control *p* < 0.0001, and between glycolytic and mito-resp ns). (**B)** H1975 cells grown orthotopically in the lungs of nude mice (*n* = 6) were dissected and analyzed using the same approach as for flank tumors (A) (mean % representation of nontargeting, glycolytic, and mito-respiratory sgRNAs are 85.4%, 61.6%, and 40.9%, respectively. One-way ANOVA of all 3 groups of sgRNAs demonstrate *p*-value = 0.006, with Tukey’s multiple comparisons test between glycolytic and control ns, mito-resp and control *p*-0.005, and between glycolytic and mito-resp ns). (**C)** Bioluminescent imaging of tumor growth and metastasis in the H1975 model (2 representative mice shown), demonstrating tumor progression in the primary site and metastatic spread to mediastinum and contralateral lung by day 28. In addition, an extrathoracic distant metastasis (left femoral metastasis) developed (blue signal in the leg of the animal on the right). All metastases were confirmed at necropsy and analyzed by sequencing. (**D)** Mean % representation of nontargeting, glycolytic, and mito-respiratory sgRNAs in H1975 metastases (7 individual metastases from 4 mice, normalized to the matched primary tumor) are plotted on the y-axis (mean and SEM shown, mean % representation of nontargeting, glycolytic, and mito-respiratory sgRNAs are 183%, 192%, and 177%, respectively, one-way ANOVA of all 3 groups of sgRNAs with Tukey’s multiple comparisons test demonstrate ns). **(E-G)** Validation of individual sgRNAs indicates that silencing mito-translational genes suppresses in vivo tumor growth. sgRNAs targeting top mito-respiratory hits identified from the mini-CRISPRi library screen—*c14orf2*, *MALSU1*, and *TMEM261*—were transduced into HCC827 cells, cells were selected with antibiotics, then injected into the flanks of nude mice. Tumors were allowed to grow for 28 days. (**E**) qPCR analysis of transduced HCC827 cells assessing level of silencing achieved with single sgRNAs (mean and SEM shown, *t* test **p < 0.01). (**F**, **G**) Tumor weight (**F**) and tumor volume (**G**) of HCC827 cells expressing each of the sgRNAs, mean and SEM shown (Student *t* test, *p < 0.05, **p < 0.01, ***p < 0.001). Underlying data can be found in S1 Data.

We postulated that similar to in vitro growth, individual gene silencing in vivo could confer relative growth advantages or disadvantages that could be estimated on the basis of sgRNA representation. After antibiotic selection, HCC827 cells were injected into the flanks of nude mice. Injected mice developed subcutaneous flank tumors that were allowed to grow for 28 days, then were analyzed by targeted sequencing as described previously [6]. We compared the normalized sgRNA representation among control nontargeting sgRNAs, sgRNAs targeting glycolytic and glycolysis-promoting genes, and sgRNAs targeting mito-ribosomal and respiratory chain (termed mito-respiratory) genes (Fig 1A). As a group, sgRNAs targeting mito-respiratory genes were significantly depleted compared to nontargeting control sgRNAs and sgRNAs targeting glycolytic genes. In fact, among all sgRNAs in the library, some individual sgRNAs targeting mito-respiratory genes, namely *MALSU1*, *HSD17β10*, *c14orf2*, and*TMEM261*, were the most significantly depleted individual sgRNAs (Fig 1A). Each of these genes is associated with mitochondrial function, and they were previously found to be critical in maintaining mitochondrial-derived ATP levels, although none have known roles in tumorigenesis. *MALSU1* (mitochondrial assembly of ribosomal large subunit 1) encodes a mitochondrial protein that is thought to be involved in mitochondrial translation [9]. *HSD17β10* encodes Hydroxysteroid 17-Beta dehydrogenase 10, which localizes to the mitochondria and is involved in protein synthesis [10]. *TMEM261* (also known as *DMAC1*) encodes transmembrane protein 261, an electron transport chain component [11]. *c14orf2* (chromosome 14 open reading frame 2, gene ATP5MPL) encodes ATP synthase membrane subunit j [12]. In a prior CRISPRi screen in HCC827 cells [6], we found that suppression of *c14orf2*, *HSD17β10*, *TMEM261*, or *MALSU1* was associated with low ATP levels in cells grown under respiratory conditions, and with high ATP levels in cells grown in glycolytic conditions (documented in Supplementary Data 4 of [6]), indicating similar ATP effects among all these genes in HCC827 cells.

The depletion of individual sgRNAs in flank tumors after 28 days of in vivo growth may reflect cellular loss in response to metabolic pressures developing not only postinjection but at any time during the tumor growth period. We assessed early in vivo tumors for changes in sgRNA representation in a separate experiment in which HCC827 cells expressing the mini-library were grown as flank tumors for either 4 or 7 days (Panel A of Fig A in S1 File). Although control sgRNAs and glycolytic gene-targeting sgRNAs (approximately 95%) were comparably expressed across these time points, mito-translation and respiratory chain sgRNAs, including the most significantly depleted sgRNAs noted above, demonstrated significantly increased expression at day 4 (Panel A of Fig A in S1 File), before becoming significantly reduced in representation at day 7, preceding the near-complete depletion measured at day 28 (Fig 1A). As a group, mito-respiratory sgRNAs demonstrated greater representation at day 4 relative to day 0 compared to control sgRNAs and glycolytic sgNAs (Panel B of Fig A in S1 File), neither of which significantly changed in this initial timeframe. These measured fluctuations in mito-respiratory sgRNAs at early time points in tumor establishment suggest shifting metabolic requirements as tumors grow. The initial increased sgRNA representation (day 4) followed by reduction at day 7 may suggest an increasing reliance on mitochondrial function as tumors grow, perhaps due to an increased requirement for mitochondrial derived ATP as the tumor establishes itself.

To assess an alternate human tumor line and in vivo tumor model, we transduced the same mini-CRISPRi sgRNA library into H1975 human lung cancer cells expressing luciferase, which grow in both flank and orthotopic lung cancer models. In separate experiments, H1975 were injected in the flanks of nude mice or into the left lower lung lobes of C.B-17 SCID mice. Tumors in both models were grown for 28 days before dissection and analysis by sequencing.

Analysis of sgRNA representation in H1975 tumors from the flank model demonstrated depletion of mito-respiratory sgRNA compared to control sgRNAs (Fig B in S1 File), similar to our findings in HCC827 cells, although the difference in mean representation of the mito-respiratory sgRNAs and the level of significance were reduced compared to HCC827 cells grown in the flank.

As an alternative site of tumor growth, H1975 tumors were also injected into an orthotopic lung tumor model. Similar to flank tumors, primary orthotopic lung tumors (Fig 1B) also demonstrated significant reduction of mito-respiratory sgRNAs as a group and severe depletion of individual *MALSU1*, *HSD17β10*, *c14orf2*, *TMEM261* sgRNAs. Orthotopic tumors in general develop in anatomically and physiologically distinct contexts that differ from subcutaneously grown flank tumors. However, these data show that primary tumors across cell lines and anatomic sites exhibit a strong requirement for mitochondrial function. Not only is this finding contrary to expectations, these data also specifically support a functionally significant need for mitochondrial-derived ATP, sharply contrasting with our findings in culture where respiratory chain genes were dispensable for growth [6].

### Mito-translational genes support in vivo tumor growth in primary sites but are dispensable in metastases

The orthotopic lung tumor model also robustly produces intrathoracic and distant metastases (Fig 1C). Tumors form at the primary site (orthotopically) then metastasize to mediastinal lymph nodes, the contralateral lung (Fig 1C) as well as distant sites, recapitulating the lethal pattern of disease spread in patients with lung cancer [13,14]. We then analyzed whether ATP-modulating genes correlate with metastatic tumor spread produced by collecting regional (mediastinal), contralateral lung, and distant metastases from the orthotopic model mice [13] (Fig 1C). Interestingly, while sgRNAs targeting mitochondrial ribosomal and respiratory chain genes were severely depleted in orthotopic tumors (Fig 1B), these sgRNAs were not depleted in metastases from the same animals (Fig 1D). As groups, control, glycolytic and mito-respiratory sgRNAs demonstrated comparable representation in metastases and were not statistically significantly different. These data suggest that ATP-modulating genes have differential effects in primary and metastatic tumors, as knocking down mitochondrial genes that are essential for primary tumor growth was dispensable for metastatic tumor deposits in the same mouse model.

Two mice developed metastases in distinctly separate anatomic compartments, and we compared sgRNA representation between these 2 sets of anatomically separated metastases, correlating across functional classes. In the first set of separate site metastases (contralateral lung and bone metastases, Panel B of Fig B in S1 File) the representation of nontargeting sgRNAs and Other Mito sgRNAs correlated (*p* < 0.01), while in the second set of separate site metastases (contralateral lung and mediastinal metastases) the nontargeting sgRNAs, glycolytic and respiratory chain sgRNAs significantly correlated (*p* < 0.05). While expanded analyses are clearly needed to fully interrogate the metabolic programs involved in metastatic progression, the correlations in this limited analysis suggest that metastases at distinct anatomic sites share some metabolic programs.

### Expressing individual sgRNAs against mito-respiratory hits suppresses in vivo tumor growth

The most depleted sgRNAs in the HCC827 flank screen and the H1975 orthotopic screen—those targeting *c14orf2*, *MALSU1*, and *TMEM261*—were prioritized for subsequent individual sgRNA experiments. We focused on *c14orf2*, *MALSU1*, and *TMEM261*, which encode mitochondrial proteins that were previously identified as strong low ATP hits under respiratory conditions [6]; these genes are among the strongest growth repressive hits in both the flank and orthotopic tumor growth screen. CRISPRi sgRNAs targeting each of these individual genes were expressed in HCC827 cells, and after silencing was confirmed (Fig 1E), the cells were injected into the flanks of nude mice. Tumors were grown for 28 days, then measured immediately after removal (Fig 1F and 1G). Each of the CRISPRi sgRNAs targeting mito-respiratory hits significantly decreased in vivo tumor growth in the flank compared to pseudogene control sgRNA. *c14orf2*, *MALSU1*, and *TMEM261* have no known roles in supporting tumor growth; however, these data provide evidence that their functions promote tumor growth and collectively suggest that mitochondrial genes are necessary for in vivo tumor growth. We assessed the tumors for reduced mitochondrial content as a possible consequence of silencing and cause of reduced respiratory function by western blotting for the mitochondrial protein TOMM20. All tumors from each sgRNA-expressing cell line demonstrated comparable TOMM20 protein levels across all sgRNAs (Fig C in S1 File), consistent with maintained mitochondrial content in the context of the silenced mito-respiratory hits.

### Suppression of mito-respiratory hits is associated with overexpression of glycolytic genes in vitro and silencing of glycolytic genes in vivo

**Fig 2:**
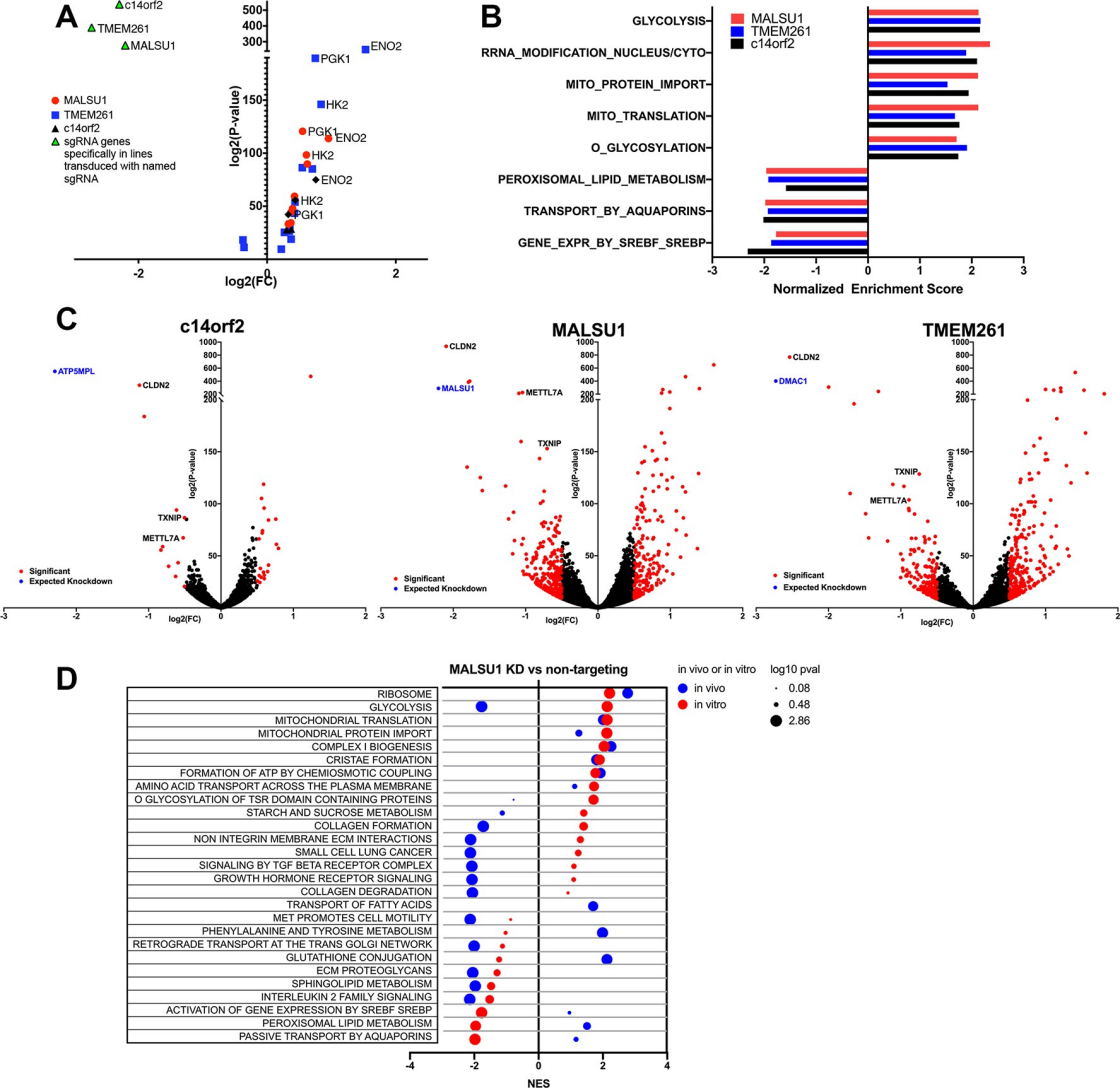
Transcriptome profiling of human lung cancer cells expressing sgRNAs against mito-respiratory hits reveals overexpression of glycolytic pathway genes in vitro and repression in vivo. RNA-Seq was performed on HCC827 cells expressing individual sgRNA against top hits *c14orf2*, *MALSU1*, *TMEM261* (or control sgRNA) (*n* = 4 samples per cell line) to determine changes in gene expression in vitro and in vivo. (**A)** Averaged expression for each mito-respiratory sgRNA was compared to control cells, fold-change, indicated by log2(FC) plotted on the x-axis, and log2(*p*-value) on the y-axis. SgRNA-targeted genes are indicated by green triangles, with each cell line demonstrating the expected reduced expression of its targeted gene (upper left quadrant). Glycolytic genes were the most overexpressed genes overall, with *ENO2*, *PGK1*, and *HK2* being the most overexpressed genes across all 3 cell lines. (**B)** Gene set enrichment analysis identifies glycolysis and mitochondria-associated pathways and ontologies as enriched in all 3 cell lines. Significance was set at *p* < 0.05. (**C)** Volcano plots shown for each of the single sgRNA cell lines. Expected sgRNA-mediated silencing was observed in all 3 cell lines (blue dot). (**D)** HCC827 cells transduced with either control sgRNA or MALSU1 sgRNA were injected into the flanks of nude mice, then grown for 28 days, after which tumors were removed and analyzed by RNA-Seq. Pathway analysis was performed comparing the *MALSU1* sgRNA cells to control sgRNA cells grown in vivo (blue) or in vitro (red), and displayed in a bubble plot indicating normalized enrichment score (NES) and log_10_ *p*-value for significantly altered pathways. Control sgRNA tumors *n* = 4, sgMALSU1 tumors *n* = 2. Underlying data can be found in S1 Data.

To gain insight into the convergent mechanisms by which these genes impact respiration, we performed RNA-Seq and transcriptional profiling analysis of HCC827 cells expressing sgRNAs against *c14orf2*, *MALSU1*, or *TMEM261*, comparing each of these to control sgRNA-expressing HCC827 cells grown under basal conditions (Fig 2A). Suppression of each of the 3 mito-respiratory sgRNAs was associated with significant overexpression of glycolytic genes (Fig 2A), with *PGK1*, *ENO2*, and *HK2* being the most significantly overexpressed glycolytic genes in all 3 analyzed lines (Fig 2A). In addition, gene set enrichment analysis identified glycolysis pathway as the most significantly altered among all 3 cell lines (Fig 2B). These concordant data support transcriptional up-regulation of glycolytic genes as a shared compensatory mechanism utilized by cancer cells when mito-respiratory function is handicapped in order to enable increased glycolytic flux.

Transcriptome profiling indicated that reactive oxygen species (ROS), glutathione, and NADPH were not consistently altered in the context of mito-respiratory gene silencing. In contrast, the expression of genes involved in fatty acid β oxidation was significantly reduced in cells in which *c14orf2*, *MALSU1*, or *TMEM261* were silenced compared to control sgRNA-expressing HCC827 cells (Fig 2B). Sterol regulatory element-binding proteins (SREBPs)-regulated gene expression, which regulate lipogenesis as well as growth and mitochondrial metabolism in some cancer cells, were also decreased upon silencing of all 3 mitochondrial genes, suggesting cross-talk between mitochondrial function and lipogenesis. Outside of the broad expression changes in major pathways, individual cell lines also demonstrated shared significantly decreased expression of *CLDN2*, *METTL7A*, and *TXNIP* (Fig 2C). *CLDN2* encodes claudin-2, a tight junction protein [15]. *METTL17* encodes a mitochondrial protein involved in the translation of mitochondrially encoded genes [16]. *TXNIP* is a thioredoxin-binding protein involved in redox regulation and glucose uptake that functions as a tumor suppressor gene [17,18]. None of these genes are known to interact with each other, and thus their common down-regulation in each of the mito-respiratory silenced cell lines implicates all 3 as participating in a transcriptional response triggered by decreased mitochondrial ATP levels. Other non-ATP functions were transcriptionally altered only in selected cell lines; that is, transcripts for genes involved in glutathione metabolism were significantly reduced in *MALSU1*-silenced cells.

HCC827 cells transduced with either control sgRNA or *MALSU1* sgRNA were injected into the flanks of nude mice, then grown for 28 days, after which tumors were removed and analyzed by RNA-Seq. Pathway analysis was performed to compare sgRNA control cells grown in vivo to the same cells grown in vitro (Fig D in S1 File). Tumor cells grown in vivo demonstrated increased expression of genes involved in collagen degradation, collagen chain trimerization, and ECM (Panel A of Fig D in S1 File), consistent with the involvement of these processes in in vivo tumor growth. Comparing pathway enrichment for *MALSU1* sgRNA-expressing cells to control sgRNA cells grown in vivo to those grown in vitro demonstrated significant similarities that are likely associated with *MALSU1* loss, notably in ribosome, mitochondrial translation, mitochondrial protein import, complex I biogenesis, and cristae formation (Fig 2D and Panel B of Fig D in S1 File).

However, this analysis also identified significant differences in transcriptomic response with *MALSU1*-silencing that were context-dependent. Specifically, expression of glycolysis pathway genes, while increased in vitro, was significantly reduced in *MALSU1* sgRNA cells grown in vivo (Fig 2D), supportive of the concept that tumor cells grown in vivo optimize respiratory function [6]. These differences indicate that the in vivo tumor growth context accentuates the significance of some pathways, and given that mitochondrial and respiratory-driven ATP is substrate-dependent, these differences in transcriptome profiles likely reflect tumor responses to substrate restriction.

### Silencing mito-respiratory genes suppresses TCA cycle activity

Silencing mito-respiratory hits was associated with shifts in gene expression (from respiration to glycolysis and vice versa) that occurred in a context-dependent manner (Fig 2). To determine how mito-respiratory hits *c14orf2*, *MALSU1*, and *TMEM261* alter metabolite levels and the pathways in which they function, we performed ^13^C-glucose and ^13^C-glutamine-based metabolomics analysis of HCC827 cells expressing individual CRISPRi sgRNAs against these genes. Cells expressing individual CRISPRi sgRNAs were grown under either basal or respiratory (10 mM 2DG), or glycolytic (oligomycin) conditions with either ^13^C-glucose or ^13^C-glutamine for 18 hours. Cells were then collected, metabolites extracted and analyzed by mass spectroscopy.

Metabolomics analysis demonstrated the relative differences in sources of TCA cycle metabolites, with Gln being the predominant source (Fig E in S1 File). Glc labeling resulted in relatively low percent labeling of citrate (ranging from 5% to 30%; seen in Panel A of Fig E in S1 File, right panel), whereas Gln labeling of most TCA cycle metabolites was approximately 80% or greater (Panel C of Fig E in S1 File, right panel). Overall, total amounts of TCA cycle metabolites varied between the 4 cell lines, with Glc and Gln labeling demonstrating similar differences between cell lines under both basal and 2DG conditions (Fig E in S1 File). Basal growth of *TMEM261* and *MALSU1*-silenced cells was associated with significant reduction in the percent labeling of the TCA metabolites citrate and aconitate (Panel A of Fig E in S1 File), while *c14orf2*-slienced cells resembled control cells. Growth under glycolytic block (2DG) generally decreased ^13^C incorporated into these TCA metabolites when compared to basal conditions, as expected, but in this condition, c14orf2-silenced cells showed reduced labeling of citrate and aconitate compared to control cells. *c14orf2* silencing reduced Glc-labeling of citrate when 2DG was present, similar to silencing *TMEM261* and *MALSU1*, indicating a deficiency shared upon silencing all 3 mito-respiratory-hits (Panel B of Fig E in S1 File, right panel). However, since this effect was only visible with a glycolytic block, *c14orf2* may be more dispensable than either *TMEM261* and *MALSUI* when glucose is available. ^13^C-glutamine-labeling also distinguished metabolite labeling profiles for cells in which mito-respiratory hits were silenced. Compared to basal control conditions, under forced respiration (2DG; Panels C and D of Fig E in S1 File, right panels), ^13^C incorporation into succinate was the most significantly reduced in all 3 mito-respiratory-deficient lines compared to control.

### Silencing mito-respiratory hits shifts cells to greater glycolytic metabolism in vitro

Genetically mediated modulation of cellular ATP involves genes that concurrently optimize 1 metabolic pathway while suppressing the alternative pathway [6]. To detect metabolic shifts towards glycolytic or respiratory function, we compared isotopologues of glycolytic and respiratory metabolites among mito-respiratory-deficient cells, grown under basal or forced respiratory (2DG) conditions (Fig F in S1 File). Examining fructose 1,6-bisphosphate (F16BP) as an index glycolytic metabolite, under basal conditions, the unlabeled (0 carbon) F16BP in *MALSU1* sgRNA and *c14orf2* sgRNA cells was comparable to control and modestly decreased in *TMEM261* sgRNA cells, while fully labeled (6 carbon) F16BP was comparable between control and *TMEM261-* as well as *c14orf2*-silenced cells and modestly reduced in *MALSU1-*silenced cells (Panel A of Fig G in S1 File, upper panels).

However, adding 2DG revealed that glycolytic metabolism in mito-respiratory-deficient cells diverged from control cells (Panel A of Fig G in S1 File, lower panels). In the presence of 2DG, all 3 mito-respiratory silenced cell lines increased labeling of the downstream glycolytic metabolite F16BP (6 carbon labeled), in contrast to control cells (Panel A of Fig G in S1 File, lower right panel), consistent with increased relative glycolytic capacity compared to control cells.

We similarly compared Gln-labeled glutamate among the cell lines under basal and forced respiratory conditions (Panel B of Fig G in S1 File). Under basal conditions, silencing of *c14orf2*, *MALSU1*, or *TMEM261* resulted in a marked increase in the percentage of unlabeled glutamate compared to control cells (Panel B of Fig G in S1 File). *c14orf2*-silenced cells had reduced fully labeled glutamate (5 carbons labeled) in contrast to *TMEM261* or *MALSU1*-silenced cells (Panel B of Fig G in S1 File, right panel), which had increased fully labeled glutamate. When grown in 2DG, *c14orf2*, *MALSU1*, and *TMEM261*-silenced cells all demonstrated increased unlabeled glutamate and reduced fully labeled glutamate compared to control cells (Panel B of Fig G in S1 File). These Gln-derived labeling patterns common to the mito-respiratory-deficient lines presumably reflect their discrete defects in respiratory metabolism that are notably worsened with forced respiration.

**Fig 3:**
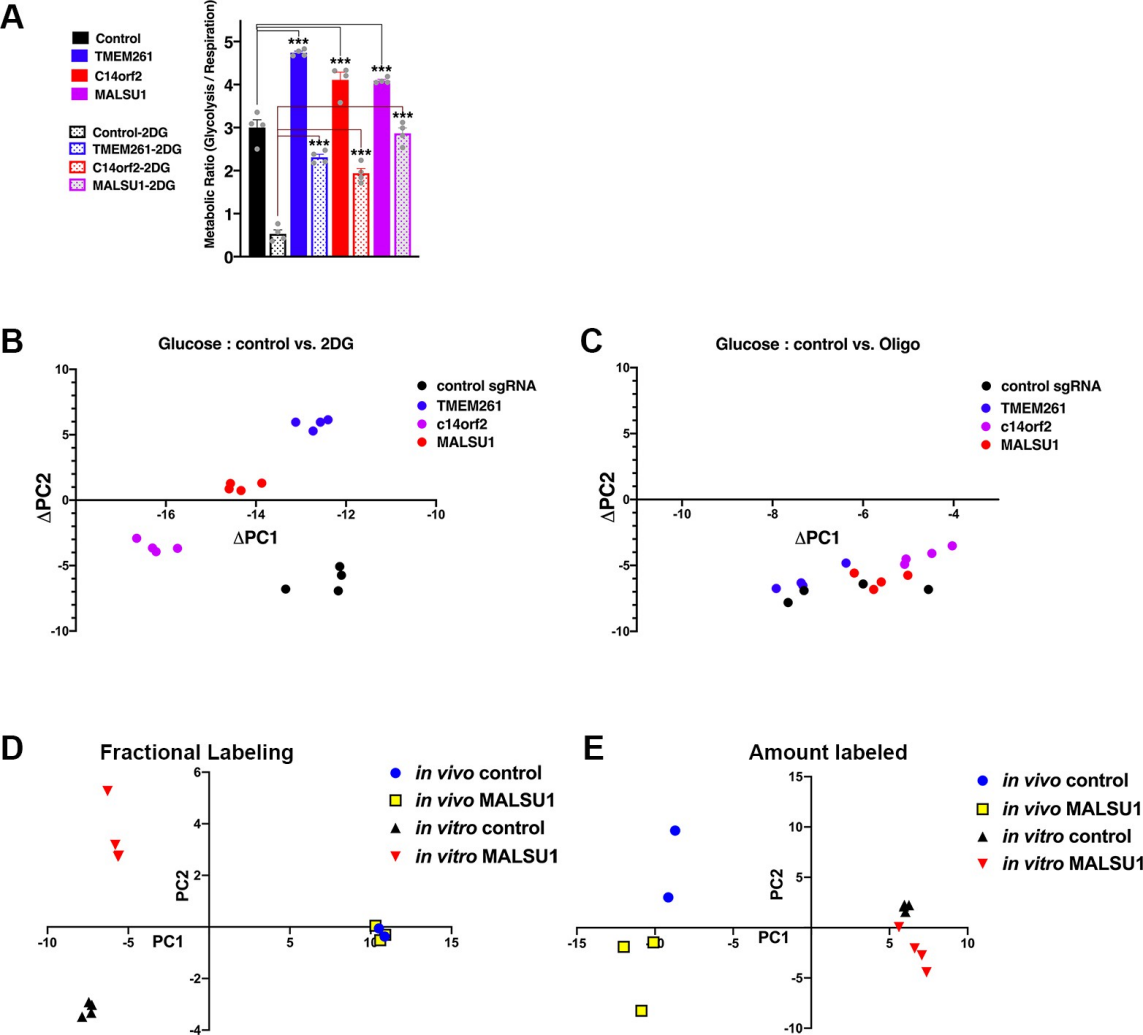
Mito-respiratory hits have distinct metabolic signatures that differ between in vitro and in vivo contexts. HCC827 cells expressing individual CRISPRi sgRNA (control, or mito-respiratory hits *c14orf2*, *MALSU1*,*TMEM261* silencing confirmation shown in Fig 2A) were grown in basal media or media with 10 mM 2DG (*n* = 4 per group, individual data points shown as grey dots) with either [U-^13^C]glucose or [U-^13^C]glutamine for 18 hours. Cells were collected, metabolites extracted and analyzed by mass spectrometry. (**A)** Estimated metabolic flux ratios for cells expressing sgRNAs targeting *c14orf2*, *MALSU1*, or *TMEM261*. Using the measured % of total all labeled and unlabeled glycolytic [[U-^13^C]glucose→F16BP indicates % total unlabeled (0 carbon) or completely labeled (6 carbon)] and respiratory metabolite values [[U-^13^C]glutamine→glutamate analysis indicates total unlabeled (0 carbon labeled) on the left and fully labeled (all 5 carbons labeled)] (shown in Panels A and B of Fig F in S1 File), the estimated metabolic flux ratios of glycolytic flux (6C/unlabeled) to the respiratory flux (5C/unlabeled) are shown for 3 mito-respiratory hits (*TMEM261*, *c14orf2*, and *MALSU1*). The ratios distinguish the glycolysis-shifted metabolism apparent in the 3 cell lines in which mito-respiratory hits are silenced. (*n* = 4 replicates per sample, 1-way ANOVA, Dunnett’s multiple comparisons test, *p < 0.05, ***p < 0.001). (**B, C)** PCA was applied to the fractional contribution values of the metabolomics data for HCC827 cells expressing individual CRISPRi sgRNA (control, or mito-respiratory hits *c14orf2*, *MALSU1*, or *TMEM261*). ^13^C glucose-derived labeling of cells grown under either control or 2DG media (D) was compared in (**B**) control or oligomycin in (**C)**. The absolute change in PC1 and PC2 values of the fractional contribution analysis for each cell line comparing control to 2DG growth (ΔPC1 = PC1_control_ − PC1_2DG_, ΔPC2 = PC2_control_ − PC2_2DG_) shown in (**B)** or control to oligomycin shown in (**C)** are plotted (*n* = 4 replicates). (**D, E)** HCC827 cells expressing either control or *MALSU1* sgRNA were injected into the flanks of nude mice. After 28 days of growth, mice were injected with ^13^C glucose to label tumor metabolites, after which tumors were collected and metabolites analyzed. (**D)** PCA was performed using the fractional labeling values of glucose-derived metabolites comparing *MALSU1*-deficient and control HCC827 cells grown in vitro or in vivo. **(E)** PCA was performed on the amount labeled of glucose-derived metabolites in MALSU1-deficient and control HCC827 cells grown in vitro or in vivo. Underlying data can be found in S1 Data. *c14orf2*, chromosome 14 open reading frame 2; F16BP, fructose 1,6-bisphosphate; *MALSU1*, mitochondrial assembly of ribosomal large subunit 1; PCA, principal component analysis.

We next used the incorporation of Glc and Gln-labeled metabolites as proxies for the utilization of glycolysis and respiration. The ratios of glycolytic label incorporation (6C/unlabeled F16BP) to the respiratory label incorporation (5C/unlabeled glutamate) for *c14orf2*, *MALSU1*, and *TMEM261*-silenced cells indicated a shared glycolysis-shifted metabolic response (Fig 3A, Panels E and F of Fig E in S1 File, and Fig F in S1 File).

Thus, while the absolute magnitude of basal Glc and/or Gln-labeling in individual glycolytic and respiratory metabolites varied between each of the mito-respiratory-silenced cell lines, the relative incorporation patterns from combined Glc and Gln-labeling highlight increased glycolytic utilization upon *c14orf2*, *MALSU1*, or *TMEM261* knockdown.

### Silencing of ATP-modulating mito-respiratory genes is associated with discrete metabolite profiles and metabolic network structure that distinguish in vitro and in vivo growth

We then examined metabolic networks on a broader scale by performing principal component analysis (PCA) of the metabolomics data (Fig 3, Figs G and H in S1 File). After computing Principal Component (PC)1 and PC2 scores for control and mito-respiratory silenced HCC827 tumor cells that were grown under either control versus 2DG, or control versus oligomycin (Figs E and G in S1 File), we plotted the ΔPC1:ΔPC2 score between control and 2DG for each cell line (Fig 3B). This depicts the net magnitude and directionality of PC score changes between control and 2DG, which did not indicate a common respiration-driven metabolite signature among the mito-respiratory silenced lines. In contrast, oligomycin treatment (forced glycolysis) was associated with similar net shifts in PC1 and PC2 scores among the 4 cellular genotypes (Fig 3C), suggesting a common glycolytic response to suppression of all 3 mito-respiratory hits (Fig 3B).

Considering the differential requirement for mito-respiratory hits in vitro versus in vivo, we next assessed metabolites in vivo. We injected HCC827 cells expressing either control or *MALSU1* sgRNA into the flanks of nude mice, grew tumors for 28 days, then injected mice with ^13^C glucose, after which tumors were collected and metabolites analyzed. We performed PCA based on the fractional contributions (amount of metabolite labeled by ^13^C glucose divided by the total metabolite pool size) and also the total amounts of measured metabolites (Fig 3D and 3E). This dimensionality reduction technique identifies PCs (PC1, PC2) from linear recombinations of the data that explain a majority of the variance. This approach allows us to compare differences in variance across fractional contributions and total amounts between conditions. These comparative analyses showed clear growth context dependence (in vitro versus in vivo) of metabolites in general, however also indicated *MALSU1*-specific effects. PC1 describes most of the variation between metabolite pool sizes and fractional labeling (62.06% and 75.52% of the variation, respectively) in vitro versus in vivo, which may result from differences in substrate introduction and metabolite extraction. However, PC2 describes variation in metabolite pool sizes and fractional labeling (16.42% and 7.7% of the variation, respectively) unassociated with in vitro versus in vivo difference, likely attributable to *MALSU1* knockdown. On the basis of fractional contributions, samples from *MALSU1* knockdown cells and control cells clustered in vivo but separated in vitro (Fig 3D). Conversely, PCA of metabolite amounts separated samples from *MALSU1* knockdown cells and samples from control cells in vivo but only marginally in vitro (Fig 3E), suggesting that *MALSU1*-silencing effects on the metabolic network in vivo are driven by discrete metabolites.

**Fig 4:**
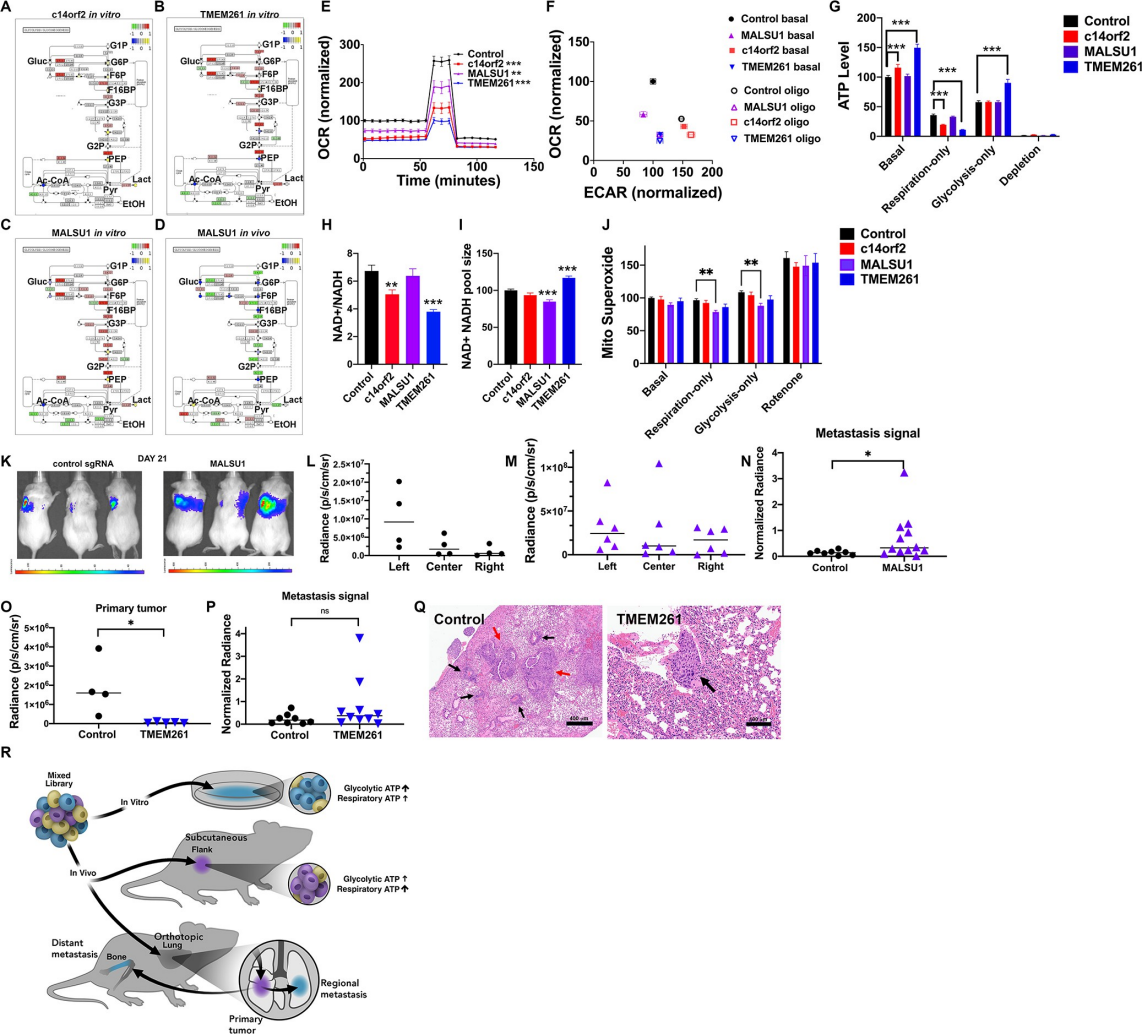
Mito-respiratory gene silencing alters metabolic network structures and in vivo tumor growth. **(A-D**) Pathway analysis of HCC827 cells expressing individual CRISPRi sgRNA (control or mito-respiratory hits *c14orf2*, *MALSU1*, or *TMEM261*) grown in vitro (**A**-**C**) or HCC827 cells expressing an individual CRISPRi sgRNA against *MALSU1* grown in vivo as flank tumor (**D**) was performed by integrating transcriptome and metabolomics data and plotted on a KEGG graph depicting pathway components for glycolysis/gluconeogenesis. (**E)** H1975 cells transduced with either control sgRNA or individual sgRNAs against *c14orf2*, *MALSU1*, or *TMEM261* were analyzed using Seahorse. OCR shown, compilation of 3 independent experiments, each analyzing *n* = 4 per cell line, normalized to cell numbers. Mean shown, 2-way ANOVA, **p < 0.01, ***p < 0.001. (**F)** OCR and ECAR measurements for cells grown under basal or forced glycolysis (oligomycin). Compilation of 3 independent experiments, each analyzing *n* = 4 replicates per cell line and normalized to cell numbers. (**G**) ATP levels were measured in different substrate conditions (basal, respiratory (10 mM 2DG), glycolytic (5 μM oligomycin) or depletion (no glucose or pyruvate)) mean and SEM shown, 2-way ANOVA, ***p < 0.001). (**H, I)** NAD+/NADH ratios and NAD+/NADH pool size. Shown are compilation of 2 independent experiments **p < 0.01, ***p < 0.001. (**J**) MitoSox assay measuring mitochondrial superoxide. Compilation of 3 independent experiments, each analyzing *n* = 4 replicates per cell line. Two-way ANOVA, **p < 0.01. (**K**) Cells expressing either control sgRNA or *MALSU1* sgRNA were injected orthotopically into the left lungs of mice, then mice were imaged using BLI 21 days postinjection. (**L)** Day 21 radiance within the left 1/3, central 1/3, and right 1/3 chest regions in mice injected with control sgRNA tumor cells. *N* = 4 mice, mean shown. (**M**) Day 21 radiance within left 1/3, central 1/3, and right 1/3, chest regions in mice injected with *MALSU1* sgRNA tumor cells. *N* = 6 mice, mean shown. (**N**) Central 1/3 and contralateral right 1/3 lung radiance each normalized to the matched left 1/3 radiance for control and *MALSU1* mice, mean shown, *t* test *p < 0.05. (**O**) Day 21postinjection radiance within the left 1/3 chest in mice injected with either control or *TMEM261*-silenced tumor cells (*n* = 4 mice for control, *n* = 5 mice for TMEM261, mean shown Mann–Whitney test, *p < 0.05). (**P**) Day 28 postinjection central 1/3 and contralateral right 1/3 lung radiance, each reading normalized to the matched left 1/3 radiance for control and TMEM261 cells (*n* = 4 mice and *n* = 5 mice, respectively) mean shown, Mann–Whitney test, ns. (**Q)** HE-stained sections of right lungs from mice injected with either control sgRNA tumor cells or TMEM261 sgRNA tumor cells showing micrometastatic tumor deposits. Red arrows indicate intraparenchymal micrometastases; black arrows indicate perivascular metastases. (**R)** Systems-level testing implicates context-specific growth effects of mitochondrial/respiratory function and ATP levels. ATP-modulating CRISPRi hits grown in lung cancer cells produce specific energy substrate-driven growth effects in vitro and in vivo. In vitro growth correlates with glycolytic ATP, while in vivo primary growth in the subcutaneous and orthotopic lung setting correlate with mitochondrial-derived ATP. Silencing discrete mito-respiratory genes also impacts the growth of regional metastases, which are distinguishable from the primary tumor. Underlying data can be found in S1A–S1D and S2E and S2F and S3G–S3J Data. BLI, bioluminescence imaging; ECAR, extracellular acidification rate; HE, hematoxylin–eosin; OCR, oxygen consumption rate.

We then sought to assess metabolic network structure for each of the mito-respiratory genes by integrating transcriptomic and metabolomic data on metabolism pathway-focused graphs [19–21] (Fig 4). All 3 mito-respiratory-deficient cell lines shared similar activation of nodes in glycolytic metabolism (Fig 4A–4C) and similar reduction in TCA cycle network (Fig I in S1 File); these shared features suggest a conserved metabolic network structure among mito-respiratory functional deficits. Furthermore, similar integrated transcriptomic and metabolomic-based pathway analysis of glucose-labeling by *MALSU1*-deficient cells grown in vivo revealed a striking reduction in glycolytic metabolite pools as compared to in vitro growth (Fig 4D), consistent with the transcriptional changes summarized in Fig 2D. These data provide further evidence that glycolytic function distinguishes the metabolic networks of mito-respiratory-deficient cells and may underlie the differential substrate-driven requirement of these genes in vivo and in vitro.

### Silencing mito-respiratory genes decreases respiration

H1975 human lung cancer cells expressing control sgRNA or sgRNA against *c14orf2*, *MALSU1*, or *TMEM261* were then compared for differences in respiratory chain function. Under basal growth conditions, silencing of all 3 mito-respiratory genes was associated with a reduction in basal and maximal oxygen consumption rate (OCR), with silencing of *TMEM261* or *c14orf2* producing more profound and significant reduction in respiration than *MALSU1* silencing (Fig 4E and 4F). Silencing of all 3 mito-respiratory genes was also associated with a reduction in ATP-linked respiration (shown as reduced shift along the y-axis with oligomycin as compared to control cells; Fig 4F). The glycolytic reserve capacity (shift in extracellular acidification rate (ECAR) along the x-axis with oligomycin) associated with all 3 mito-respiratory hits was similarly reduced versus control. *c14orf2* silencing also resulted in elevated baseline glycolysis. We then measured ATP levels in cells across multiple substrates (basal, respiratory, glycolytic) and found that under basal conditions, *c14orf2* and *TMEM261* silencing are associated with increased ATP (Fig 4G). Under respiratory conditions, *c14orf2* and *TMEM261* silencing significantly reduced ATP (Fig 4G). Under glycolytic conditions, ATP was similar across lines with the exception of *TMEM261*, whose silencing was associated with increased ATP (Fig 4G). Among all the mito-respiratory hits, *TMEM261* silencing was associated with the largest ATP fluctuations across conditions, suggesting that *TMEM261* function may be more substrate-dependent than *c14orf2* and *MALSU1*. Interestingly, *MALSU1* silencing was not associated with changes in ATP levels, although OCR was reduced compared to control, presumably reflecting a compensatory reduction in ATP consumption to equilibrate against reduced ATP production.

To determine potential effects of mito-respiratory gene silencing on redox function, we then measured NAD+/NADH levels (Fig 4H and 4I). Silencing of either *c14orf2* or *TMEM261* significantly decreased NAD(H) pool size compared to control sgRNA (that is, NAD+ and NADH), while NAD(H) pool size was unchanged in *MALSU1*-silenced cells. For *TMEM261*-silenced cells, the reduction in NAD+/NADH ratio occurred in the context of a significantly increased total NAD(H) pool size, whereas *MALSU1*-silenced cells demonstrated a slightly reduced NAD(H) pool size compared to control cells. Taken together, these data further support decreased oxidative phosphorylation activity for *c14orf2* and *TMEM261*-silenced cells but indicate that the mito-respiratory genes have distinct effects on NAD+/NADH metabolism, suggesting that changes in NAD+/NADH metabolism do not underlie their effects on tumor growth and survival. The observed changes in NAD+/NADH metabolism may indicate that there are changes in NAD+/NADH synthetic or catabolic processes less directly related to energy metabolism that occur in *MALSU1* or *TMEM261*-silenced cells. In summary, changes in NAD+/NADH metabolism were not consistent between mito-respiratory silenced lines and therefore are unlikely to explain the similar changes in growth.

Mitochondrial production of ROS is a by-product of respiration, and excessive ROS production can influence cell growth. To determine whether mito-respiratory gene-silencing results in ROS overproduction, which can be a limiting factor in tumor cell growth, we measured mitochondrial superoxide levels in mito-respiratory gene-silenced cells across multiple substrates (basal, respiratory, and glycolytic). Mitochondrial superoxide levels were only modestly reduced in cells expressing sgRNA against *MALSU1*, and comparable to control in the other mito-respiratory-deficient cell lines across all 3 substrates (Fig 4J), arguing against an increase in mitochondrial ROS potentially causing toxicity to cells and inhibiting tumor growth by this mechanism.

### Silencing of individual mito-respiratory genes increases metastatic progression

To test whether mito-respiratory hits impact in vivo growth of primary and/or metastatic tumors, H1975 cells expressing luciferase, dCas9-KRAB, and control sgRNA, *MALSU1* sgRNA, or *TMEM261* sgRNA were injected orthotopically into the left lungs of nude mice. In the orthotopic tumor model, bioluminescence imaging (BLI) signal first develops in the primary site, then develops in the mediastinum and the contralateral lung (Fig 4K and 4L). Mice injected with *MALSU1*-silenced cells developed rapidly progressive and more extensive BLI signal in regions distal from the primary site (in the central/mediastinal or contralateral chest) than mice injected with control sgRNA cells (Fig 4K–4N). This finding is consistent with overall more rapid tumor establishment in vivo and may or may not indicate unique effects on metastases. To compare the proportional metastatic disease burden relative to the primary tumor, we normalized the BLI signals of the mediastinal and also contralateral lung regions to the matched primary tumor. Interestingly, compared to control sgRNA, *MALSU1* silencing was associated with increased metastatic BLI signal relative to the primary tumor (Fig 4N). Tumor cells in which *TMEM261* was silenced demonstrated significantly slower overall disease progression and reduced primary tumor growth compared to control tumor cells (Fig 4O). However, despite their suppressed primary tumor growth, *TMEM261*-silenced tumors still generated central and contralateral pulmonary metastases that were comparable to control sgRNA tumors in BLI signal and morphology (Fig 4P and 4Q). These data indicate that metastatic progression was maintained in *TMEM261*-silenced tumor cells and suggest that primary tumors contrast with metastases in their requirement for intact *TMEM261* function, supporting a model of metabolic heterogeneity.

In summary, individual silencing of either *MALSU1* or *TMEM261* each significantly impacted tumor progression in vivo, with differential effects on primary and metastatic progression, revealing that tumors in different locations differ in their metabolic requirements (Fig 4R). In *TMEM261*-silenced tumors, primary tumor growth was suppressed, which contrasts with *MALSU1*-silenced tumors. This difference may be linked to the larger ATP fluctuations across basal, glycolytic, to respiratory metabolism that occurred with *TMEM261* silencing. *TMEM261* silencing was associated with the most severe reduction in respiration and ATP among all the mito-respiratory hits (Fig 4E and 4F), and the suppression of primary tumor growth (as compared to control tumors and *MALSU1*-silenced tumors) correlates with reduced respiratory ATP. A shared finding for *TMEM261* and *MALSU1* silencing is the preservation of metastatic progression, which in the case of *MALSU1* was notably accelerated. These data indicate that metastatic progression in our model occurs independent of primary tumors reaching a certain size, and that suggest that respiratory ATP is needed for primary tumor establishment but not metastases.

## Discussion

Previous studies have implicated cell-intrinsic metabolic differences as important determinants of metastatic potential [22]. However, primary tumors and subsequent metastases occupy diverse anatomic locations, and growth in different sites may require alternate metabolic processes specific to the local environment [23]. These locally defined requirements and the metabolic capabilities tumor cells must possess to negotiate them remain to be defined but would have significant clinical and therapeutic implications if understood. Metastatic disease remains essentially fatal and causes serious morbidity in cancer patients, and, therefore, improving the control of any subset of metastases would likely be beneficial for patients.

Lung cancers in particular are known to utilize multiple metabolic pathways to support growth in vitro [24]; however, which metabolic pathways are required for in vivo growth of primary and metastatic lung cancer is not well understood. In our study, human lung cancer cells were grown in multiple in vivo contexts to test whether genes modulating ATP were selectively required. The enrichment and depletion of select classes of genes implicated specific metabolic requirements for in vivo tumor growth. The contrasts in functional outcome between in vitro and in vivo tumor growth suggest that tumor cells utilize aerobic respiration in a context-dependent manner to meet ATP requirements in vivo, rather than being hard-wired to preferentially utilize aerobic glycolysis at all times.

*c14orf2*, *MALSU1*, and *TMEM261* were top CRISPRi ATP-modulating hits that each underlie respiratory chain dysfunction, severely impacting ATP production [6] as well as in vivo tumor growth. The mechanisms by which each of these genes accomplishes this effect are not fully known. We previously found that silencing another respiratory ATP-modulating gene, *HSD17β10*, was associated with a slow rate of ATP depletion under respiratory metabolism but a rapid rate of ATP depletion under basal substrate conditions [6]. This example indicates that mechanisms promoting either ATP production or ATP consumption can each contribute to ATP levels and metabolic phenotype in disease-defining ways. *c14orf2*, which is part of ATP synthase/complex V, *MALSU1* (needed for normal translation or function of mitochondrial ribosomes), and *TMEM261* (a component of complex I) are mitochondrial proteins, although there are no known functional connections between these genes and how they act on respiration.

Metabolomics analysis and transcriptional profiling of the 3 mito-respiratory hits identified a unified transcriptional and metabolomic signature that notably shifts glycolytic utilization. Importantly, the effect of *c14orf2*, *MALSU1*, and *TMEM261* silencing on the expression of glycolysis pathway genes depended on the environmental context, resulting in significant glycolysis pathway reduction in vivo, but not in vitro, and only the latter case is consistent with the Warburg effect.

Our analysis of mitochondrial function supports respiratory ATP reduction, rather than ROS or NAD+ depletion, as underlying the cell growth effects of silencing mito-respiratory hits. All 3 cell lines we examined had deficient baseline respiration, maximal respiration, and ATP-linked respiration relative to control cells, but had divergent ROS or NAD+/NADH-related phenotypes. *c14orf2* and *TMEM261* silencing decreased NAD+/NADH ratio, which could indicate impaired NADH oxidation by the electron transport chain. Meanwhile, *MALSU1* silencing decreased the total NAD(H) pool size (NAD+ and NADH), but this metabolite pool size increased with *TMEM261* silencing. These changes could reflect shifts in the biosynthesis or use of NAD+/NADH pools for nonenergetic functions, including DNA repair or epigenetic modification [25]. Importantly, these genes could have different effects in vivo in primary tumors versus metastases, and other effects of the respiratory chain may contribute to the in vivo growth effects measured after silencing mito-respiratory hits.

In vivo, the effects of *MALSU1* and *TMEM261* silencing on tumor growth differed while demonstrating that metastasis formation relative to primary tumor was promoted. *TMEM261*-silenced cells produced a smaller primary tumor compared to control cells, but silencing either *MALSU1* or *TMEM261* resulted in increased metastasis signal relative to the primary tumor. The precise metabolic mechanisms dictating the overlapping and nonoverlapping effects of these genes are unclear, but these genes differed in multiple respects with regard to respiratory function. *TMEM261* silencing was associated with a more profound and significant reduction in respiration than *MALSU1* silencing (Fig 4E). *TMEM261* silencing was also associated with the largest ATP fluctuations across substrates (basal, glycolytic, to respiratory metabolism; seen in Fig 4G). These biochemical effects coupled with in vivo growth effects suggest that tumors modulate respiration using systems-level controls to adapt to local metabolic substrates.

The differences we observed in in vivo growth based on gene function may be evidence of metabolic plasticity on the part of tumor cells to successfully colonize different anatomic sites and metabolic environments. Tumor cells interact with their environments, and analysis of tumor microenvironments by tumor interstitial fluid analysis have quantified differences in metabolite levels between tumor locations [23]. Our data are consistent with tumor cells shifting to glycolytic metabolism when forming metastases, contrasting with primary tumor growth at an initial site of origin. This shift may serve to support increased biosynthesis needed to establish metastases and/or may occur in response to limited nutrients/oxygen. When precisely the metabolic shift occurs during tumor progression is unclear, but it may happen early in the primary tumor’s establishment, before other competing selective pressures shape metastatic potential (as can be seen in bottlenecking effects). Alternatively, or possibly in addition, local selective pressures (after initial seeding from the primary tumor) may favor metastases that are adapted to optimize glycolytic metabolism. Our work was focused on metastases within the thoracic cavity, and metastases in more distant and diverse organs could differ. Finally, cancer cells take up and secrete metabolites [7,23], influencing the cellular constituents of the microenvironment, which importantly includes immune cells. Our model utilized immunodeficient mice, which is a limitation as the immune system modulates tumor growth and is itself influenced by the tumor microenvironment [26].

In spite of these limitations and remaining questions, a unique characteristic of our model is that metastases spontaneously develop from the primary tumor, spreading initially to the mediastinum and then to the contralateral lung, which mirrors lung cancer progression in patients and correlates to clinical lung cancer staging definitions [27]. This contrasts to modeling approaches that inject tumor cells directly into the bloodstream to simulate hematogenous metastases [28], but in the process circumvent the intrinsic pathologic steps from which metastases are seeded from primary tumors. Thus, our experimental approach is designed to functionally evaluate the primary to metastasis progression in a single physiologically relevant platform.

Since metastases arise from primary tumors, they could be anticipated to mirror the sgRNA abundance of the primary tumor. Instead, we identified asymmetric representation of sgRNAs in metastases relative to the primary tumor, which could result from multiple possible mechanisms. These include dependence on respiration as a selection factor and/or changing metabolic needs of tumor cells (possibly based on their size, perfusion, and substrate availability of the microenvironment that they inhabit). Metastatic cells spreading from the primary tumor early in development (possibly prior to the primary tumor reaching a size at which specific metabolic substrates become growth-limiting) might initially resemble primary tumors in their sgRNA representation. Metastases may then diverge as local growth conditions limit energy substrates, skewing sgRNA representation. Alternatively, cells with respiratory chain dysfunction may be predisposed to metastasize early, meaning that metastases are metabolically distinct from primary tumors from the beginning. Bottlenecking effects may also significantly influence the pool of tumor cells available to metastasize and effectively skew the number of sgRNAs represented in tumors. Our analyses of early tumor time points (Panel A of Fig A in S1 File) indicate that sgRNAs are not immediately depleted after injection, but there are undoubtedly additional steps in the dynamic process of metastasis formation that should be elaborated upon in future studies.

What could account for the tumors’ heterogeneity in dependence upon mitochondrial function? Our prior work defining genetic modulators of cellular ATP indicated that many genes and pathways that preserve or reduce ATP exert these effects only under specific metabolic conditions defined by substrate availability [6]. Silencing respiratory/mitochondrial genes reduced ATP under respiratory conditions but increased ATP when cells grew under glycolytic conditions [6]. Cancer cells may use mito-respiratory genes to gain metabolic advantage when encountering different substrate environments. Our data suggest a greater role of glycolysis in metastases formation, which may be due to local perfusion, oxygen availability, or substrate availability. These possible mechanisms should be explicitly tested in future studies.

Other contributing mechanisms of metastasis, such as cellular migration and invasion, and escape from immune surveillance could invoke alternative metabolic adaptations (such as production of specific metabolites and alternate bioenergetic processes). We analyzed established in vivo metastases that had clearly completed multiple steps of metastasis formation. The scope of this initial work does not enable us to resolve the intermediate steps of metastasis formation, and thus the dependencies we observe may localize to different stages of metastases. An obvious question would also be how cancer cells transition across metabolic states. Indeed, our data strongly hint at dynamic alterations, as mito-respiratory sgRNAs significantly increased between initial injection and day 4 of flank growth, before significantly depleting between day 4 and day 7 (Panel A of Fig A in S1 File). Elucidating these steps will require appropriately tailored modeling approaches.

Our study focused on lung cancers, and it should be acknowledged that other tumor cell types may shift differently between glycolytic and respiratory metabolism, invoking other metabolic processes. For example, melanoma tumor cell survival and in vivo disease progression, including metastasis, have been shown to be facilitated by suppression of ferroptosis [29,30]. The potential diversity of metabolic phenotypes assumed by tumor cells should be experimentally elaborated and may contribute to the general goal of eradicating metastatic disease.

Metastatic disease is recognized to be genetically polyclonal and distinct from primary cancers [31]. By taking a systems-driven experimental approach into physiologic contexts, our work contributes to understanding metastases as poly-metabolic, that is, involving discrete metabolic states that are likely adapted to the local metabolism and a cell’s bioenergetic needs. This metabolic heterogeneity appears to distinguish primary and metastatic tumors, which can have significant implications for treating metastatic disease. To date, metabolism-based therapies are not routinely integrated into cancer management, although novel agents and strategies are under investigation [3,26]. Greater basic understanding of cancer metabolism to inform metabolically based treatment approaches is needed and could comprise a productive treatment regimen for patients with advanced cancers.

## Materials and methods

### Cell culture

The human lung adenocarcinoma HCC827 cell line was originally obtained from Trever Bivona (UCSF) (ATCC 2868, 39-year-old Caucasian female individual). HCC827 cells were grown at 37°C in RPMI medium with 10% FBS, 1% penicillin/streptomycin. H1975 cells expressing GFP-luciferase were grown at 37°C in DMEM medium with 10% FBS, 1% penicillin/streptomycin.

### RNA isolation, reverse transcription (RT), and real-time RT-PCR to confirm gene knockdown

RNeasy Mini Kit (Qiagen) was used to isolate total RNA. SuperScript IV (Thermo Fisher Scientific) was used to synthesize cDNA. Gene expression was measured by real-time PCR on QuantStudio 5 using Taqman probes. All Taqman probes were purchased from Thermo Fisher Scientific, and assay IDs are Beta-Actin (Hs99999903_m1), HSD17B10 (Hs00189576_m1), MALSU1 (Hs00370770_m1), TMEM261 (Hs00383923_m1), and c14orf2 (Hs01043634_m1). cDNAs and PCR reactions were prepared according to the protocol for Cells-to-CT kit (Thermo Fisher #AM1728), using the standard reverse transcription cycle (95°C for 2 min, inactivation at 95°C for 5 min, hold at 4°C), and qRT-PCR conditions (UDG Incubation– 50°C for 2 min, enzyme activation– 95°C for 10 min, PCR cycle– 95°C for 15 s, 60°C for 1 min–repeat 40 cycles). All reactions were performed in a 384-well plate, in replicates of at least *n* = 3 and from 2 independent experiments. CT (threshold cycle) values of each gene were averaged and calculated relatively to CT values of β-actin using the 2^−△△CT^ method [32].

### Flank xenografts

Surgical procedures and all animal work were done under a protocol AN182206-02, approved by the UCSF Institutional Animal Care and Use Committee (IACUC). Transduced cells were injected (1 × 10^6^ cells) into the right flank of nude mice as previously described [33]. Injected mice developed subcutaneous flank tumors that grew for 28 days and then were analyzed by sequencing. Mice were killed by carbon dioxide inhalation followed by cervical dislocation.

### Orthotopic lung xenografts in immunodeficient mice

Six- to 8-week-old female SCID CB.17 mice were obtained from Charles River and housed in pathogen-free conditions and facilities as previously described [13]. Briefly, tumor cells expressing GFP-Luc were suspended in Matrigel (Corning). Cell concentration was adjusted to 1 × 10^5^ cells/μl, and cell suspension was transferred into a 1-ml syringe. Syringe was kept on ice until implantation. After anesthetizing, a 1-cm surgical incision was made along the posterior medial line of the left thorax. A volume of 10-μl cell suspension was injected into left lung directly. Visorb 4.0 polyglycolic acid sutures were used for primary wound closure of the skin layer.

### Bioluminescence imaging

Xenogen IVIS-100 (PerkinElmer) was used for in vivo imaging. Mice were injected intraperitoneally with VivoGlo luciferin, in vivo grade: P1042(Promega) (150 mg/kg). After 10 min, mice were anesthetized with 2% isoflurane, and then they were transferred into Xenogen IVIS-100. All mice were imaged twice weekly. Living Image (PerkinElmer) was used for analysis. All imaging processes followed manufacturer’s instructions. To measure BLI signal in the left, center, and right chest, a rectangular box capturing the chest cavity of each mouse was divided into thirds of equal area that correspond to left, center, and right. Comparisons between sets of experimental mice were made using BLI quantitation made on the same day, during the same imaging session.

### Tumor and lung tissue collection following mouse orthotopic injection

Mice were killed by carbon dioxide inhalation followed by cervical dislocation. Tissues were collected from mice postmortem. Tumor tissues were snap-frozen in liquid nitrogen and stored in −80°C. Lung tissues were collected into 4% paraformaldehyde and incubated at 4°C overnight. Lung tissues were then transferred into ethanol 70%. Lung tissues were embedded into paraffin blocks, sectioned at 4 μm, and stained with hematoxylin–eosin (HE) by the UCSF Histology and Biomarker Core. HE-stained slides were scanned and analyzed histopathologically (AEH).

### In vitro metabolomics

Approximately 5 × 10^5^ cells per well were plated in 6-well plates. After 24 h, cell medium was aspirated. Cells were rinsed quickly with ice-cold 150 mM NH4AcO (pH 7.3). After removing NH4AcO, 1 ml 80% MeOH was added, which was precooled on dry ice. Plates were incubated on dry ice for 20 min. Samples were transferred into 1.5 ml tubes placed on ice, then each tube was vortexed for 10 s. After centrifugation at 16,000*g* for 15 min at 4°C, supernatants were transferred into new tubes. Samples were dehydrated in a speed vac. Samples were stored in −80°C. All samples were analyzed by the UCLA Metabolomics Center [6].

HCC827 cells were incubated for 2 and 6 h in either [1] respiratory condition: 2% FBS, 10 mM [U-^13^C]pyruvate + 10 mM 2DG, [2] glycolytic condition: 2% FBS, 2 mM [U-^13^C]glucose + 5 μM oligomycin + 3 mM 2-deoxyglycose, [3] basal condition: 2% FBS, 10 mM [U-^13^C]glucose + 5 mM pyruvate with no drugs, or [4] 2% FBS, 10 mM [U-^13^C]pyruvate alone, before metabolite extraction in 80% methanol and drying in a Labconco CentriVap. Dried metabolites were resuspended in 50% ACN:water and loaded onto a Luna 3um NH2 100 A (150 × 2.0 mm) column (Phenomenex). The chromatographic separation was performed on a Vanquish Flex (Thermo Scientific) with mobile phases A (5 mM NH4AcO, pH 9.9) and B (ACN) and a flow rate of 200 μL/min. A linear gradient from 15% A to 95% A over 18 min was followed by 9 min isocratic flow at 95% A and reequilibration to 15% A. Metabolites were detection with a Q Exactive mass spectrometer (Thermo Scientific) run with polarity switching (+3.5 kV/−3.5 kV) in full scan mode with an m/z range of 65 to 975. TraceFinder 4.1 (Thermo Scientific) was used to quantify the targeted metabolites by area under the curve, using expected retention time and accurate mass measurements (<5 PPM). Values were normalized to cell number. Relative amounts of metabolites were calculated by summing up the values for all isotopologues of a given metabolite. Metabolite isotopologue distributions were corrected for natural C13 abundance.

### In vivo metabolomics

Mice undergoing the procedure were fasted for 12 h prior to the infusion of ^13^C-glucose. The mice were weighed the morning of the procedure and anesthetized with 1.5% isoflurane (v/v). Catheters constructed from 30 g needles (BD 305106) and polyethylene tubing (BD 427400) were used to infuse a 200-μL bolus of ^13^C-glucose (0.4 mg/g) into the tail vein. Following the bolus, a 150-μL/h infusion of ^13^C-glucose at a dosage of 0.012 mg/g/min was administered for 30 min [34–36]. Mice were then killed and the tumors were flash frozen in an isopentane bath cooled to −80°C with dry ice. To extract the metabolites from the frozen tissue, the samples were first homogenized in a cryogenic mortar and pestle before being mixed with 1 mL of 80% methanol chilled to −80°C. Samples were then vortexed for 20 s and incubated at −80°C for 20 min. Following the incubation, the samples were vortexed for an additional 20 s and then centrifuged at 16,000*g* for 15 min in a 4°C chamber. The supernatant was transferred to a −80°C prechilled 1.5 mL tube. To normalize the extracted metabolite amounts by protein content, a BCA assay was completed on the sample pellets. A 100-μg protein equivalent of extracted metabolite was aliquoted from each sample and dried in a Labconco CentriVap. The dried samples were then stored at −80°C and analyzed by the UCLA Metabolomics Center.

### Western blotting

Western blotting was performed as previously described [37] using TOMM20 antibody(ab186735) (Abcam) and anti-β actin (#4967, Cell Signaling) and anti-rabbit IgG, HRP-linked secondary antibody (#7074, Cell Signaling).

### Sequencing and computation of sgRNA representation

Genomic DNA was isolated using the Macherey-Nagel NucleoBond Xtra Midi Plus (Macherey-Nagel, Germany). The sgRNAs were amplified and adaptors attached in a single PCR step. Approximately 1.5 μg of undigested genomic DNA was used per 50 μL PCR reaction, and sufficient reactions were performed to include all isolated genomic DNA. PCR was conducted using Q5 HotStart High Fidelity Polymerase (NEB, Ipswich, MA) using forward primer: aatgatacggcgaccaccgaGATCGGAAGAGCACACGTCTGAACTCCAGTCACNNNNNNgcacaaaaggaaactcaccct 1 and reverse primer: caagcagaagacggcatacgaCGACTCGGTGCCACTTTTTC, which include necessary adaptor and indexing sequences. “N” refers to the variable index sequence. PCR parameters were 98°C for 30 s, followed by 26 cycles of 98°C for 15 s, 62.5°C for 15 s, 72°C for 20 s, and ending with 72°C for 6 min; samples were then ramped down to 4°C and held. The resulting PCR product from multiple reactions were pooled, and unincorporated primers were removed using the GeneRead Size Selection Kit. Quality and purity of the PCR product was assessed by bioanalyzer (Agilent), and sequencing was performed on an Illumina HiSeq 2500 as described [38]. Informatic analysis of the raw reads was performed as described [38].

### Respiration and glycolysis measurement

ECAR (a surrogate for glycolysis) and OCR (to assess mitochondrial respiration) were measured using a 96-well Seahorse XF96 Extracellular Flux Analyzer (Seahorse Bioscience). One day before the assay, a Seahorse assay cartridge was calibrated with calibration medium in a CO_2_-free incubator. A total of 30,000 H1975 cells per well were seeded into an XF96 cell culture microplate. The next day, cells were incubated with Seahorse XF DMEM medium buffered to pH 7.4. For ECAR measurements, the media was supplemented with 10 mM glucose and 5 mM pyruvate; for OCR measurements, the media was supplemented with 10 mM pyruvate and 10 mM 2DG. Cells were incubated in this media for 1 h prior to the assay in a CO_2_-free incubator. Baseline OCR was measured, followed by measurement after sequential addition of 1 μM FCCP and 1 μM rotenone. Baseline ECAR was measured, followed by measurement after sequential addition of 1 μM oligomycin A, followed by 1 μM rotenone. After each run, cells were fixed with 4% paraformaldehyde, and the OCR and ECAR signals were normalized to the number of cells in each well, estimated using Hoescht 33342 staining.

### Biochemical assays

For ATP, mitochondrial ROS, and NAD+/NADH measurements, 20,000 H1975 cells were seeded per well in a 96-well plate. One day later, cells were treated with PBS supplemented with 2% FBS along with 5 mM pyruvate + 10 mM glucose (basal), 10 mM pyruvate and 10  mM 2DG (respiratory), or 2 mM glucose, 5 μM oligo and 3 mM 2DG (glycolytic) for 1 h. For ATP measurements, an additional treatment of 10 mM 2DG and 5 μM oligo was included as an ATP-depleted control condition. ATP measurements were performed using the Promega CellTiterGlo 2.0 kit. For mitochondrial superoxide measurements, cells were first stained with 2.5 μM MitoSOX Red for 15 min at 37°C prior to 1 h treatments with the above substrates. An additional treatment of 2 μM rotenone was included as a high ROS control condition. For NAD+ and NADH measurements, 1% dodecyltrimethylammonium bromide solution was used to lyse cells, before splitting samples into a 0.4 N HCl-treated fraction and untreated fraction. Samples were heated at 60°C for 15 min, before neutralizing acid with Trizma base. NAD+ and NADH levels were measured in the acid and nonacidified fractions, respectively, using the Promega NAD/NADH-Glo Assay. All luminescence and fluorescence were measured on a Spectramax M4 plate reader.

### RNA-Seq

RNA was isolated from pellets of approximately 1 million cells using an RNeasy mini kit with DNase set (Qiagen). RNA sequencing was performed by Novogene (https://en.novogene.com). Sequencing reads were analyzed in Galaxy [39], using the FastQC tool for quality control [40]. HISAT2 was used to align reads against the homo sapiens b37 hg19 genome [41]. Read counts were quantified using the FeatureCounts tool [42]. Differentially expressed genes were identified using DESeq2 [43]. Pathway analysis was performed on sequenced transcripts preranked by fold-change in expression using FGSEA [44]. Transcriptomic data were integrated with metabolomic data by inputting lists of fold-change or percent change for gene expression and metabolite amount or percent labeling into Pathview [20,45], which was then used to integrate metabolomic and transcriptomic data, with log2FC of genetic knockdowns versus controls as the input. For compound data input, log2FC of metabolite pool sizes versus controls were averaged across all replicates. For gene input, log2FC of expression level versus controls were averaged across all replicates.

### Gene function and pathway analyses

Preranked gene set enrichment analysis [46,47] was used to determine enriched pathways and ontology terms among high and low ATP genes. The gene list was collapsed to unique gene identifiers, and were ranked based on the magnitude of their ATP phenotype. The maximum gene set size was set at 500 genes, and the minimum size at 10 genes. One thousand random sample permutations were carried out using the Molecular Signature Database c2 v6.2 and c5 v6.2, and a significance threshold was set at a nominal *p*-value of 0.05.

### Principal component analysis

The R function prcomp was used for PCA, with fractional labeling or amount labeling of metabolites as the input, and unit scaling. The first and second PC were plotted for each analysis, corresponding to the 2 components that explain the most variance in the data.

### Quantification and statistical analysis

All statistical analyses, including the n, what n represents, description of error bars, statistical tests used, and level of significance, are stated in the figure legends. All measurements were taken from distinct samples. Prism 9 (GraphPad) was used for statistical calculations.

## Supporting information

S1 File **Fig A. sgRNA representation in flank tumors at early time points. (**A) HCC827 cells transduced with the mini-CRISPRi library were injected into the flanks of nude mice, then grown for either 4 or 7 days (Day 4 or Day 7), after which tumors were removed and analyzed by sequencing and read count analysis. Cells at injection are Day 0 samples. Number of replicates: Day 0 *n* = 3, Day 4 *n* = 3, Day 7 *n* = 2. sgRNA representation for selected sgRNAs (negative control sgRNAs, a subset of sgRNAs that were enriched in the initial flank experiment (Day 28) (*PAF1*, *DIRAS1*, *SCO1*, *HK3*), and depleted sgRNAs in the flank experiment (*HSD17B10* (3 individual sgRNAs), *MALSU1*, *HJURP*, *TMEM261 and c14orf2*) are plotted. Mean and SEM shown for the labeled sgRNA at each time point (Day 0, Day 4, and Day 7). Student *t* test, two-sided performed for comparison of named sgRNA at each time point, * *p* < 0.05, **p < 0.01, ***p < 0.001. Respiratory sgRNA representation are similar to nonrespiratory control and glycolytic sgRNAs initially, enrich at Day 4 or 7 relative to nonrespiratory sgRNAs. (B) The relative change from Day 0 to Day 4 in sgRNA representation in classes of sgRNAs. sgRNA read counts for HCC827 flank tumors at Day 4 were normalized to Day 0 sgRNA read count. Mean for each group indicated by line. One-way ANOVA with multiple comparisons Tukey’s test (*p < 0.05). Underlying data can be found in S1 Data. **Fig B**. **sgRNA representation in H1975 tumors**. (A) H1975 human *EGFR*-mutant lung cancer cells were transduced with the mini-CRISPRi library and then were injected into the subcutaneous space in flanks of nude mice and grown for 28 days. DNA from each tumor was sequenced and read counts for each sgRNA quantified. The read count for each sgRNA was normalized to negative control sgRNAs and the ratio of each sgRNA’s frequency in the tumor model relative to its frequency in vitro (immediately preinjection). H1975 cells grown as flank tumors were analyzed, *n* = 6. Each dot represents a single sgRNA and indicates its average normalized representation displayed as control sgRNAs and sgRNAs targeting ATP-modulating genes classified as glycolytic or mito-respiratory (termed Mito-Resp). (Mean (red line) and SEM shown, mean % representation of nontargeting, glycolytic, and mito-respiratory sgRNAs are 92.5%, 116.3%, and 75.0%, respectively. One-way ANOVA of all 3 groups of sgRNAs demonstrate *p*-value = 0.03). (B) sgRNA frequencies in a lung metastasis are plotted on the x-axis against the identical sgRNA frequencies in the bone metastasis from the same animal. Dots representing individual sgRNAs are color coded by classification as nontargeting sgRNA, glycolytic, mitochondrial protein synthesis (Mito Protein Synth), respiratory chain (Resp Chain), and other mitochondrial function (Other Mito). Underlying data can be found in S1 Data. **Fig C. Mitochondrial protein level in HCC827 cells expressing mito-respiratory sgRNAs grown as flank tumors**. Whole cell lysates were prepared from individual flank tumors (*n* = 6 flank tumors of each sgRNA–control sgRNA (sgCont), *c14orf2*, *MALSU1*, *TMEM261*). Immunoblotting for the mitochondrial membrane protein TOMM20 was performed, with β-Actin as loading control. **Fig D. Pathway analysis of mito-respiratory silenced cells.** HCC827 cells transduced with control sgRNA were injected into the flanks of nude mice, then grown for 28 days, after which tumors were removed and analyzed by RNA-Seq (*n* = 4 tumors analyzed). (**A)** Gene set enrichment analysis was performed to compare control expression profiles in sgRNA-expressing cells grown in vivo to in vitro. (**B)** The expression profile for each *MALSU1* sgRNA tumor replicate (Rep 1 and Rep 2, red and blue bars, respectively) was analyzed, compared to control sgRNA and enriched/depleted pathways plotted (with normalized enrichment score). The replicates are concordant in pathway enrichment across most pathways, with the exception of mitochondrial protein import and amino acid transport across the plasma membrane. Underlying data can be found in S1 Data. **Fig E. Relative levels and fractional labeling of glycolytic and TCA metabolites in mito-respiratory-silenced cells.** HCC827 cells expressing individual CRISPRi sgRNA (control, or mito-respiratory hits *c14orf2*, *TMEM261*, *MALSU1*, silencing confirmation shown in Fig 2A), were grown in basal media or media with 10 mM 2DG (*n* = 4 per group) with either [U-^13^C]glucose or [U-^13^C]glutamine for 18 h. Cells were collected and metabolites analyzed. (**A)** [U-^13^C]glucose-labeled TCA intermediates (relative levels at left and % labeled at right) demonstrates that under basal conditions, silencing of *TMEM261* and *MALSU1* is associated with significantly reduced citrate and aconitate (right panel, 1-way ANOVA *p < 0.05, **p < 0.01, ***p < 0.001, ****p < 0.0001). (**B)** Forced respiration (2DG) reduces glucose-derived labeling of TCA intermediates, as expected. Mito-respiratory-silenced cells demonstrate significantly reduced citrate and aconitate compared to control cells. (**C, D)** [U-^13^C]glutamine labeling of cells under basal (**C**) and respiratory (2DG) (**D**) conditions (relative levels at left and % labeled at right). Under basal conditions, *TMEM261* demonstrates increased [U-^13^C]glutamine-derived labeling of acetyl-coA compared to control cells, whereas *c14orf2* cells demonstrate decreased [U-^13^C]glutamine-derived labeling of acetyl-coA compared to control cells. When 2DG is added, % labeled acetyl-coA is reduced all mito-respiratory cell lines (in the case of *TMEM261* cells, this reduction is relative to the elevated level under basal metabolism (**C**). In addition, all 3 mito-respiratory lines develop highly significant reductions in succinate labeling (right panel, *p < 0.05, **p < 0.01, ***p < 0.001, ****p < 0.0001). (**E, F)** Relative levels of F16BP (**E**) and glutamate (**F**) are shown in cells grown without or with 2DG. One-way ANOVA with Dunnett’s multiple comparisons test *p < 0.05, **p < 0.01, ***p < 0.001. Underlying data can be found in S1 Data. **Fig F. Isotopologues for F16BP and glutamate**. (**A**) All labeled isotopologues for ^13^C glucose➔ F16BP indicates % total unlabeled (0 carbon) or completely labeled (6 carbon) (*n* = 4 replicates per sample, mean and SEM shown). This data relates to Fig 3A. B. All labeled isotopologues for ^13^C glutamine➔ glutamate indicates % total unlabeled (0 carbon) or completely labeled (5 carbon) (*n* = 4 replicates per sample, mean and SEM shown). This data relates to Fig 3A. Underlying data can be found in S1 Data. **Fig G. Metabolomics and principal component analysis.** HCC827 cells expressing individual CRISPRi sgRNA (control, or mito-respiratory hits *TMEM261*, *c14orf2*, *MALSU1*, silencing confirmation shown in Fig 2A), were grown in basal media or media with 10 mM 2DG (*n* = 4 per group, individual data points shown as grey dots) with either [U-^13^C]glucose or [U-^13^C]glutamine for 18 h. Cells were collected, metabolites extracted and analyzed by mass spectrometry. (**A**) [U-^13^C]glucose➔ fructose 1,6-bisphosphate indicates % total unlabeled (0 carbon) or completely labeled (6 carbon) with top graphs indicating percent total and fractional labeling (left and right, respectively) under basal conditions and the bottom graphs indicating percent total and fractional labeling in the presence of 2DG (*n* = 4, mean, SEM). Respiratory-deficient cells demonstrate relative resistance to 2DG, maintaining glycolytic activity and labeling in the presence of 2DG (shown in bottom right panel, 1-way ANOVA, Dunnett’s multiple comparisons test, *p < 0.05, **p < 0.01, ***p < 0.001, NS, not significant). (*n* = 4 replicates per sample, mean and SEM shown). (**B**) [U-^13^C]glutamine➔ glutamate analysis indicates total unlabeled (0 carbon labeled) on the left and fully labeled (all 5 carbons labeled) on the right. Control cells maintain glutamine uptake (left panel) after addition of 2DG, which is not expected to affect respiration. Compared to control, respiratory-deficient cells demonstrate reduced uptake of glutamine (reflected in increased total unlabeled) at baseline (left panel). Adding 2DG exaggerates this defect in glutamine uptake by respiratory-deficient cells (reflected in increased % unlabeled, shown in left panel). In the right panel, U-^13^CGlutamine➔ glutamate indicates activity through TCA. 2DG increases glutamine metabolism through TCA in control cells, consistent with compensatory metabolic shift to TCA and intact TCA. Basally, respiratory-deficient cells (*TMEM261* and *MALSU1*) demonstrate a modest but significant increase in TCA metabolism compared to control sgRNA cells (right panel), which we hypothesize represents a compensatory drive to increase activity through TCA to accommodate the respiratory defect. However, 2DG overcomes this compensation and further exacerbates the TCA defect in respiratory-deficient cells, which demonstrate significantly and severely decreased metabolism through TCA. (**C, D**) Principal component analysis was applied to the fractional contribution values of the metabolomics data for HCC827 cells expressing individual CRISPRi sgRNA (control, or mito-respiratory hits *TMEM261*, *MALSU1*, or *c14orf2*. ^13^C-glucose-derived labeling of cells grown under either control or 2DG media (C) or control or oligomycin in (D). The PC1 and PC2 values of the fractional contribution analysis is plotted for each cell line grown under either control or 2DG conditions (*n* = 4 replicates for each line) is plotted. Underlying data can be found in S1 Data. **Fig H. Skreeplots of principal component analysis of** ^**13**^**C-glucose or** ^**13**^**C-glutamine metabolomics.** Principal component analysis (PCA) was performed to assess similarities in metabolite responses among the mito-respiratory cell lines. All 4 cell lines (control sgRNA and 3 mito-respiratory lines) were pooled to analyze either ^13^C-glucose or ^13^C-glutamine metabolites under control conditions and either 2DG or oligomycin. Two principal components explain the majority of the variance for all 4 comparisons. Underlying data can be found in S1 Data. **Fig I**. **Integrated metabolomics and transcriptomics-based pathway analysis distinguishes metabolic network structure among mito-respiratory cells.** Pathway analysis was performed by integrating transcriptome and metabolomics data for HCC827 cells expressing individual CRISPRi sgRNA (control or mito-respiratory hits *c14orf2*, *TMEM261* or *MALSU1*) grown in vitro and plotted on a KEGG graph depicting pathway components for citrate cycle (TCA cycle). Underlying data can be found in S1 Data. **Fig J. Quantitative PCR shows silencing of mito-respiratory hits in H1975 cells.** H1975 cells transduced with individual sgRNAs against *c14orf2*, *MALSU1*, or *TMEM261* were analyzed by qPCR to confirm silencing of the targeted gene. *N* = 4 samples per cell line for each probe, mean and SEM shown.
(PDF)

S1 Data Data sheet index below 1. **In vitro and in vivo growth screen data, HCC827 cells, Source data for Fig 1.** Mini-library CRISPRi screen in HCC827 cells, raw data. The read counts for each sgRNA in all in vitro and in vivo (flank and orthotopic) replicates are included. **2. In vitro and in vivo growth screen data, H1975 cells, Source data for Fig 1.** Mini-library CRISPRi screen in H1975 cells, raw data. The read counts for each sgRNA in all in vitro and in vivo (flank and orthotopic) replicates are included. **3. Metastases Source data. Source data for Fig 1. 4. Time course. Source data for S1 Fig. 5. Tumor weight and volume, Source data for Fig 1. 6. Metabolomics C**^**13**^**-Glucose, Isotopologue Source data for Fig 3. 7. Metabolomics C**^**13**^**-Glutamine, Isotopologue Source data for Fig 3. 8. Metabolomics C**^**13**^**-Glucose, Fractional Contribution Source data for Fig 3. 9. Metabolomics C**^**13**^**-Glutamine, Fractional Contribution Source data for Fig 3. 10. RNA-Seq in vitro neg vs. in vitro sgc14orf2, Source data for Fig 2**. **11. RNA-Seq in vitro neg vs. in vitro sgMALSU1, Source data for Fig 2. 12. RNA-Seq in vitro neg vs. in vitro sgTMEM261, Source data for Fig 2. 13. RNA-Seq in vivo neg vs. in vivo sgMALSU1, Source data for Fig 2D. 14. Pathway analysis of c14orf2, MALSU1, TMEM261 15. Bioluminescence data. Source data for Fig 4.** Radiance readings and normalized values for replicates are listed here.
(XLSX)

S2 Data Seahorse readings. (XLSX)

S3 Data ATP, NADH, and Mitosox Data readings. (XLSX)

S1 Raw Images Raw images for Fig C in S1 File. (PDF)

## Abbreviations

BLI: bioluminescence imaging
CT: threshold cycle
c14orf2: chromosome 14 open reading frame 2
EGFR: epidermal growth factor receptor
F16BP: fructose 1,6-bisphosphate
HE: hematoxylin–eosin
MALSU1: mitochondrial assembly of ribosomal large subunit 1
PC: principal component
PCA: principal component analysis
ROS: reactive oxygen species
SREBP: sterol regulatory element-binding protein
