# Supplementary material for: Primary and metastatic tumors exhibit systems-level differences in dependence on mitochondrial respiratory function
Source: PLoS Biol. 2022 Sep 22;20(9):e3001753. doi: 10.1371/journal.pbio.3001753 (PMC9498964; doi:10.1371/journal.pbio.3001753)

Fig A. sgRNA representation in flank tumors at early time points. A. HCC827 cells transduced with the mini-CRISPRi library were injected into the flanks of nude mice, then grown for either four or seven days (Day 4 or Day 7), after which tumors were removed and analyzed by sequencing and readcount analysis. Cells at injection are Day 0 samples. Number of replicates: Day 0 n=3, Day 4 n=3, Day 7 n=2. sgRNA representation for selected sgRNAs (negative control sgRNAs, a subset of sgRNAs that were enriched in the initial flank experiment (day 28) (*PAF1, DIRAS1, SCO1, HK3*), and depleted sgRNAs in the flank experiment (*HSD17B10* (3 individual sgRNAs), *MALSU1*, *HJURP*, *TMEM261 and c14orf2*) are plotted. Mean and s.e.m. shown for the labeled sgRNA at each timepoint (Day 0, Day 4 and Day 7). Student's t-test, two sided performed for comparison of named sgRNA at each time point, * p<0.05, **p<0.01, ***p<0.001. Respiratory sgRNA representation are similar to non-respiratory control and glycolytic sgRNAs initially, enrich at day 4 or 7 relative to non-respiratory sgRNAs. B. The relative change from day 0 to day 4 in sgRNA representation in classes of sgRNAs. SgRNA read counts for HCC827 flank tumors at Day 4 were normalized to Day 0 sgRNA readcount. Mean for each group indicated by line. 1 way ANOVA with multiple-comparisons Tukey's test (*p< 0.05).

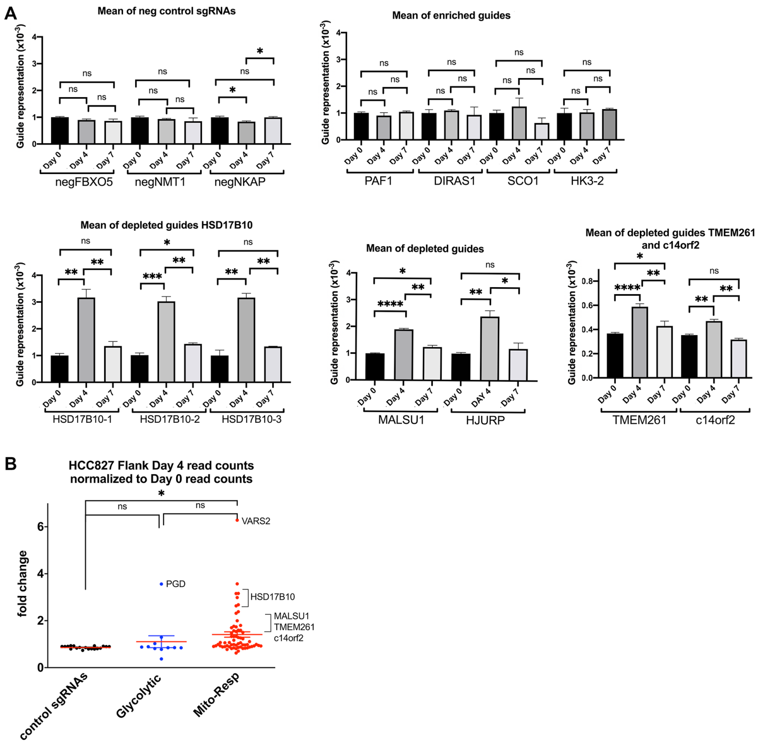

Fig B. **sgRNA representation in H1975 tumors**. A. H1975 human *EGFR*-mutant lung cancer cells were transduced with the mini-CRISPRi library and then were injected into the subcutaneous space in flanks of nude mice and grown for 28 days. DNA from each tumor was sequenced and read counts for each sgRNA quantified. The read count for each sgRNA was normalized to negative control sgRNAs and the ratio of each sgRNA's frequency in the tumor model relative to its frequency *in vitro* (immediately pre-injection). H1975 cells grown as flank tumors were analyzed, n=6. Each dot represents a single sgRNA and indicates its average normalized representation displayed as control sgRNAs and sgRNAs targeting ATP-modulating genes classified as glycolytic or mito-respiratory (termed Mito-Resp). (Mean (red line) and s.e.m. shown, mean % representation of non-targeting, glycolytic and mito-respiratory sgRNAs are 92.5%, 116.3% and 75.0% respectively. One-way ANOVA of all three groups of sgRNAs demonstrate p value=0.03). B. SgRNA frequencies in a lung metastasis are plotted on the x-axis against the identical sgRNA frequencies in the bone metastasis from the same animal. Dots representing individual sgRNAs are color coded by classification as non-targeting sgRNA, glycolytic, mitochondrial protein synthesis (Mito Protein Synth), respiratory chain (Resp Chain) and other mitochondrial function (Other Mito).

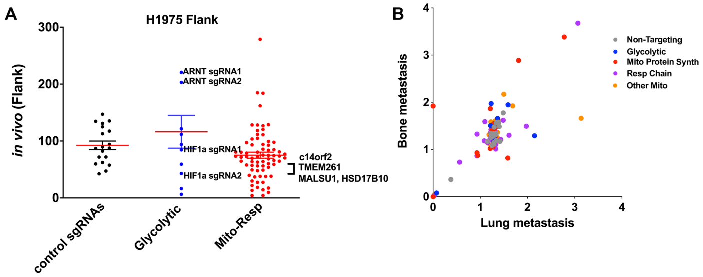

Fig C. Mitochondrial protein level in HCC827 cells expressing mito-respiratory sgRNAs grown as flank tumors. Whole cell lysates were prepared from individual flank tumors (n=6 flank tumors of each sgRNA – control sgRNA (sgCont), *c14orf2*, *MALSU1*, *TMEM261*). Immunoblotting for the mitochondrial membrane protein TOMM20 was performed, with β-Actin as loading control.

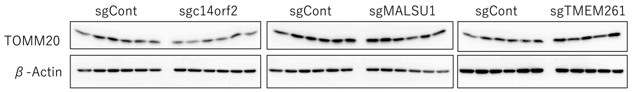

Fig D. Pathway analysis of mito-respiratory silenced cells. HCC827 cells transduced with control sgRNA were injected into the flanks of nude mice, then grown for 28 days, after which tumors were removed and analyzed by RNA Seq (n=4 tumors analyzed). **A.** Gene set enrichment analysis was performed to compare control expression profiles in sgRNA-expressing cells grown *in vivo* to *in vitro*.

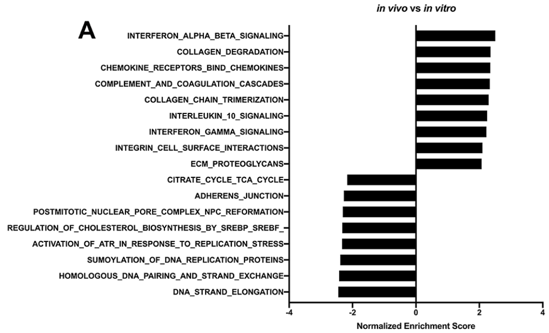

B. The expression profile for each *MALSU1* sgRNA tumor replicate (Rep 1 and Rep 2, red and blue bars respectively) was analyzed, compared to control sgRNA and enriched/depleted pathways plotted (with normalized enrichment score). The replicates are concordant in pathway enrichment across most pathways, with the exception of mitochondrial protein import and amino acid transport across the plasma membrane.

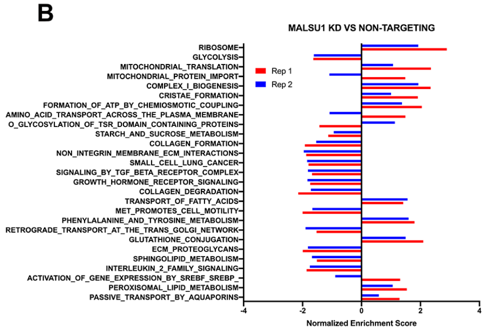

Fig E. Relative levels and fractional labeling of glycolytic and TCA metabolites in mito-respiratory-silenced cells. HCC827 cells expressing individual CRISPRi sgRNA (control, or mito-respiratory hits *c14orf2*, *TMEM261*, *MALSU1*, silencing confirmation shown in Fig. 2A), were grown in basal media or media with 10 mM 2DG (n=4 per group) with either U-^{13}C]glucose or [U-^{13}C]glutamine for 18 hours. Cells were collected and metabolites analyzed. **A.** [U-^{13}C]glucose labeled TCA intermediates (relative levels at left and % labeled at right) demonstrates that under basal conditions silencing of *TMEM261* and *MALSU1* is associated with significantly reduced citrate and aconitate (right panel, 1-way ANOVA *p<0.05, **p<0.01, ***p<0.001, ****p<0.0001). **B.** Forced respiration (2DG) reduces glucose-derived labeling of TCA intermediates, as expected. Mito-respiratory-silenced cells demonstrate significantly reduced citrate and aconitate compared to control cells. **C/D.** [U-^{13}C]glutamine labeling of cells under basal (C) and respiratory (2DG)(D) conditions (Relative levels at left and % labeled at right). Under *basal* conditions, *TMEM261* demonstrates increased [U-^{13}C]glutamine-derived labeling of acetyl-coA compared to control cells, whereas *c14orf2* cells demonstrate decreased [U-^{13}C]glutamine-derived labeling of acetyl-coA compared to control cells. When 2DG is added, % labeled acetyl-coA is reduced all mito-respiratory cell lines (in the case of *TMEM261* cells, this reduction is relative to the elevated level under basal metabolism (C). In addition, all three mito-respiratory lines develop highly significant reductions in succinate labeling (right panel, *p<0.05, **p<0.01, ***p<0.001, ****p<0.0001). **E/F.** Relative levels of F1,6BP (E) and glutamate (F) are shown in cells grown without or with 2DG. One-way ANOVA with Dunnett's multiple comparisons test *p<0.05, **p<0.01, ***p<0.001.

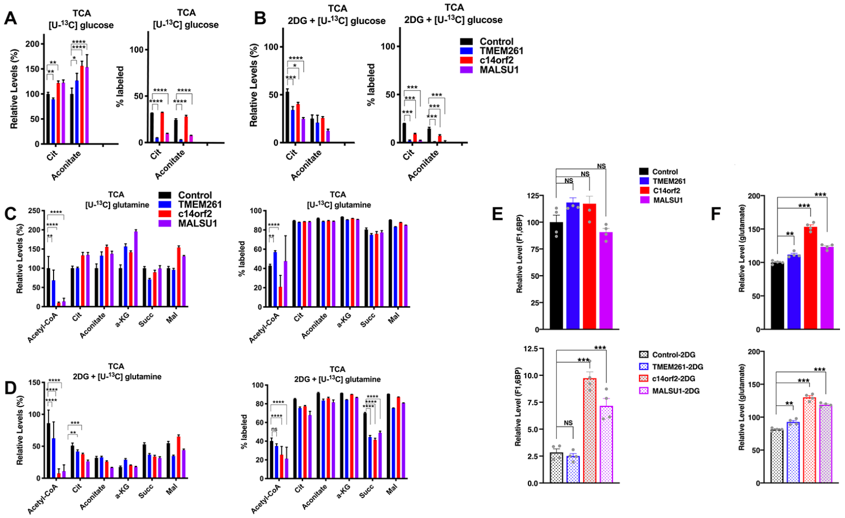

Fig F. Isotopologues for F16BP and Glutamate. A. All labeled isotopologues for ^{13}C glucose→ F16BP indicates % total unlabeled (0 carbon) or completely labeled (6 carbon) (n=4 replicates per sample, mean and s.e.m. shown). **B.** All labeled isotopologues for ^{13}C glutamine→ Glutamate indicates % total unlabeled (0 carbon) or completely labeled (5 carbon) (n=4 replicates per sample, mean and s.e.m. shown).

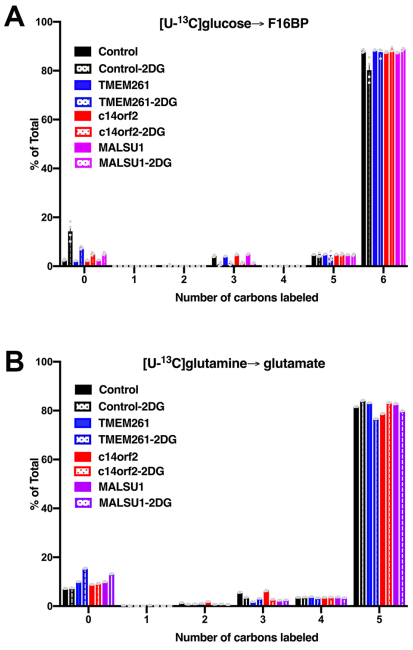

Fig G. Metabolomics and principal component analysis
Legend on next page.

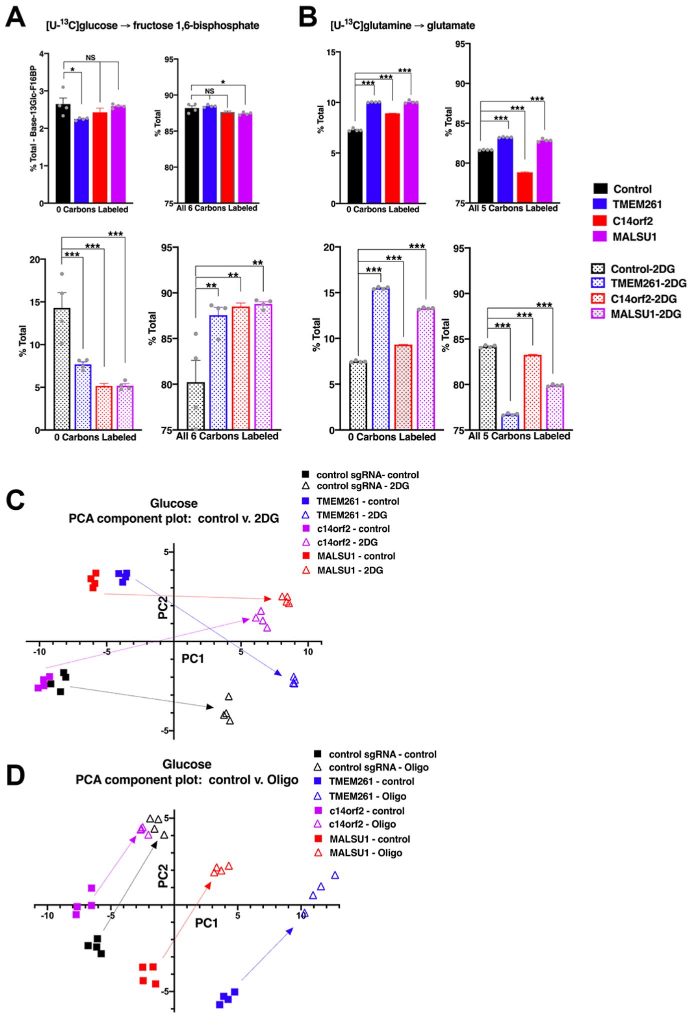

Fig G. Metabolomics and principal component analysis. HCC827 cells expressing individual CRISPRi sgRNA (control, or mito-respiratory hits *TMEM261, c14orf2*, *MALSU1*, silencing confirmation shown in Fig. 2A), were grown in basal media or media with 10 mM 2DG (n=4 per group, individual data points shown as grey dots) with either [U-^{13}C]glucose or [U-^{13}C]glutamine for 18 hours. Cells were collected, metabolites extracted and analyzed by mass spectrometry. **A**. [U-^{13}C] glucose→ fructose 1,6-bisphosphate indicates % total unlabeled (0 carbon) or completely labeled (6 carbon) with top graphs indicating percent total and fractional labeling (left and right, respectively) under basal conditions and the bottom graphs indicating percent total and fractional labeling in the presence of 2DG (n = 4, mean, sem). Respiratory-deficient cells demonstrate relative resistance to 2DG, maintaining glycolytic activity and labeling in the presence of 2DG (shown in bottom right panel, 1-way ANOVA, Dunnett's multiple comparisons test, *p<0.05, **p<0.01, ***p<0.001, NS-not significant). (n=4 replicates per sample, mean and s.e.m. shown). **B**. [U-^{13}C]glutamine→ glutamate analysis indicates total unlabeled (0 carbon labeled) on the left and fully labeled (all 5 carbons labeled) on the right. Control cells maintain glutamine uptake (left panel) after addition of 2DG, which is not expected to affect respiration. Compared to control, respiratory-deficient cells demonstrate reduced uptake of glutamine (reflected in increased total unlabeled) at baseline (left panel). Adding 2DG exaggerates this defect in glutamine uptake by respiratory-deficient cells (reflected in increased % unlabeled, shown in left panel). In the right panel, U-^{13}CGlutamine→ glutamate indicates activity through TCA. 2DG increases glutamine metabolism through TCA in control cells, consistent with compensatory metabolic shift to TCA and intact TCA. Basally, respiratory-deficient cells (*TMEM261* and *MALSU1*) demonstrate a modest but significant increase in TCA metabolism compared to control sgRNA cells (right panel), which we hypothesize represents a compensatory drive to increase activity through TCA to accommodate the respiratory defect. However, 2DG overcomes this compensation and further exacerbates the TCA defect in respiratory-deficient cells, which demonstrate significantly and severely decreased metabolism through TCA. **C/D**. Principal component analysis was applied to the fractional contribution values of the metabolomics data for HCC827 cells expressing individual CRISPRi sgRNA (control, or mito-respiratory hits *TMEM261*, *MALSU1*, or *c14orf2*. ^{13}C glucose-derived labeling of cells grown under either control or 2DG media (C) or control or oligomycin in (D). The PC1 and PC2 values of the fractional contribution analysis is plotted for each cell line grown under either control or 2DG conditions (n=4 replicates for each line) is plotted.

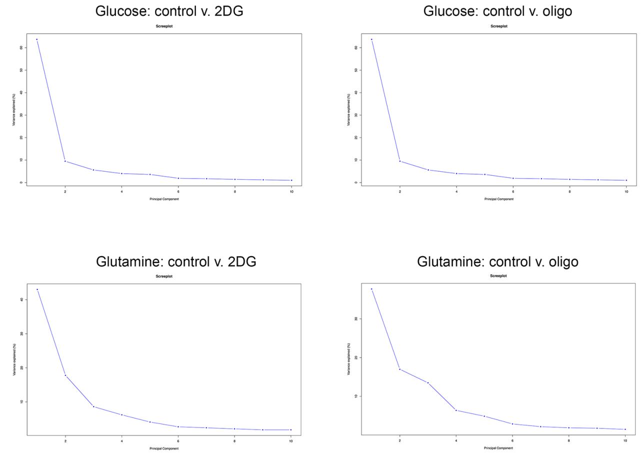

Fig H. Skreeplots of principal component analysis of ^{13}C-glucose or ^{13}C-glutamine metabolomics. Principal component analysis (PCA) was performed to assess similarities in metabolite responses among the mito-respiratory cell lines. All four cell lines (control sgRNA and three mito-respiratory lines) were pooled to analyze either ^{13}C-glucose or ^{13}C-glutamine metabolites under control conditions and either 2DG or oligomycin. Two principal components explain the majority of the variance for all four comparisons.

Fig I. Integrated metabolomics and transcriptomics-based pathway analysis distinguishes metabolic network structure among mito-respiratory cells. Pathway analysis was performed by integrating transcriptome and metabolomics data for HCC827 cells expressing individual CRISPRi sgRNA (control or mito-respiratory hits *c14orf2*, *TMEM261* or *MALSU1*) grown *in vitro* and plotted on a KEGG graph depicting pathway components for citrate cycle (TCA cycle).

A **c14orf2**

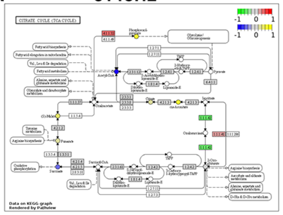

B **TMEM261**

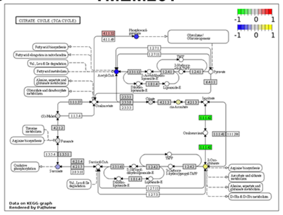

C **MALSU1**

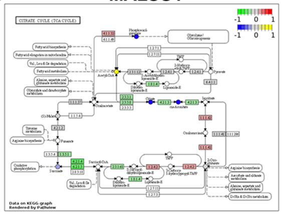

Fig J. Quantitative PCR shows silencing of mito-respiratory hits in H1975 cells. H1975 cells transduced with individual sgRNAs against *c14orf2*, *MALSU1* or *TMEM261* were analyzed by qPCR to confirm silencing of the targeted gene. N = 4 samples per cell line for each probe, Mean and s.e.m. shown.

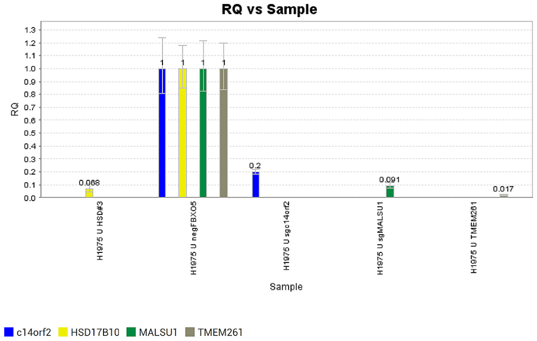

Supplement: S1 File — Fig A. sgRNA representation in flank tumors at early time points. (A) HCC827 cells transduced with the mini-CRISPRi library were injected into the flanks of nude mice, then grown for either 4 or 7 days (Day 4 or Day 7), after which tumors were removed and analyzed by sequencing and read count analysis. Cells at injection are Day 0 samples. Number of replicates: Day 0 n = 3, Day 4 n = 3, Day 7 n = 2. sgRNA representation for selected sgRNAs (negative control sgRNAs, a subset of sgRNAs that were enriched in the initial flank experiment (Day 28) (PAF1, DIRAS1, SCO1, HK3), and depleted sgRNAs in the flank experiment (HSD17B10 (3 individual sgRNAs), MALSU1, HJURP, TMEM261 and c14orf2) are plotted. Mean and SEM shown for the labeled sgRNA at each time point (Day 0, Day 4, and Day 7). Student t test, two-sided performed for comparison of named sgRNA at each time point, * p < 0.05, **p < 0.01, ***p < 0.001. Respiratory sgRNA representation are similar to nonrespiratory control and glycolytic sgRNAs initially, enrich at Day 4 or 7 relative to nonrespiratory sgRNAs. (B) The relative change from Day 0 to Day 4 in sgRNA representation in classes of sgRNAs. sgRNA read counts for HCC827 flank tumors at Day 4 were normalized to Day 0 sgRNA read count. Mean for each group indicated by line. One-way ANOVA with multiple comparisons Tukey’s test (*p < 0.05). Underlying data can be found in S1 Data. Fig B. sgRNA representation in H1975 tumors. (A) H1975 human EGFR-mutant lung cancer cells were transduced with the mini-CRISPRi library and then were injected into the subcutaneous space in flanks of nude mice and grown for 28 days. DNA from each tumor was sequenced and read counts for each sgRNA quantified. The read count for each sgRNA was normalized to negative control sgRNAs and the ratio of each sgRNA’s frequency in the tumor model relative to its frequency in vitro (immediately preinjection). H1975 cells grown as flank tumors were analyzed, n = 6. Each dot represents a single sgRNA a [file pbio.3001753.s001.pdf]
