# Supplementary figures and images for: Primary and metastatic tumors exhibit systems-level differences in dependence on mitochondrial respiratory function
Source: PLoS Biol. 2022 Sep 22;20(9):e3001753. doi: 10.1371/journal.pbio.3001753 (PMC9498964; doi:10.1371/journal.pbio.3001753)

Original Images for blot results

TOMM20

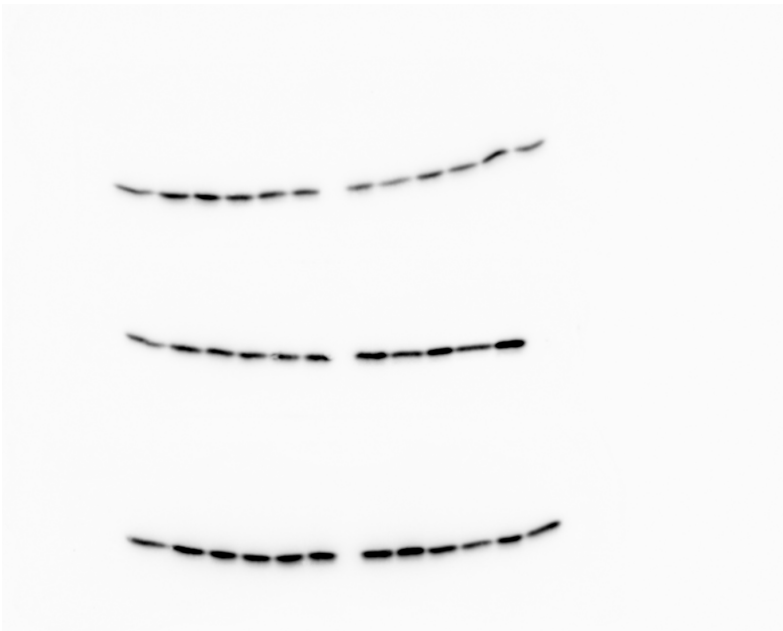

$\beta$  actin

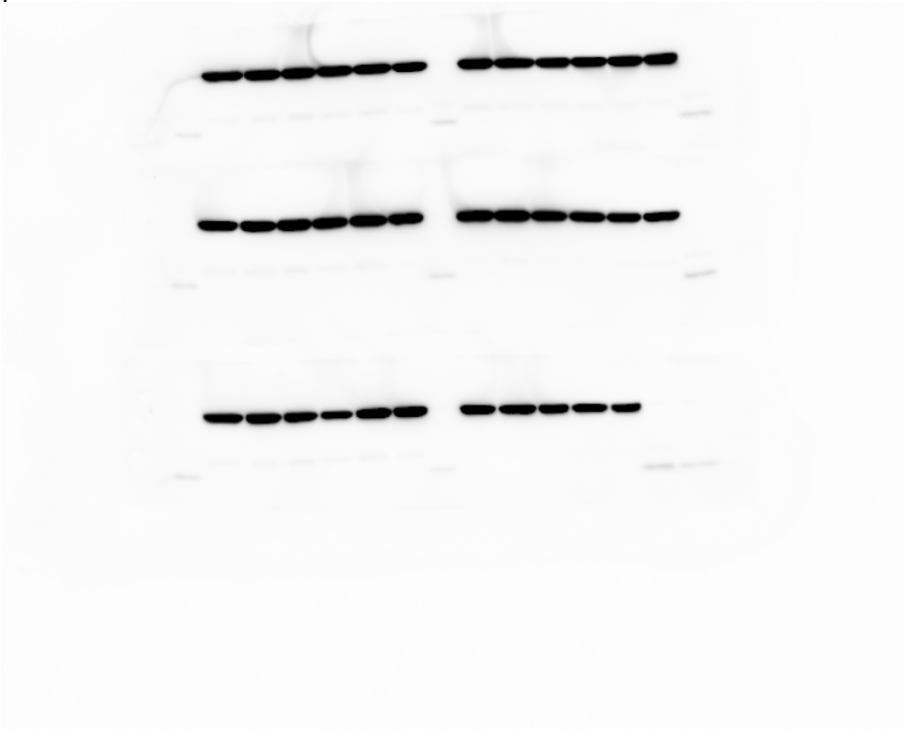

Supplement: S1 Raw Images — (PDF) [file pbio.3001753.s005.pdf]
